# HNRNPU promotes the progression of triple-negative breast cancer via RNA transcription and alternative splicing mechanisms

**DOI:** 10.1038/s41419-022-05376-6

**Published:** 2022-11-08

**Authors:** Bo-yue Han, Zhebin Liu, Xin Hu, Hong Ling

**Affiliations:** 1 Department of Breast Surgery, Fudan University Shanghai Cancer Center, Fudan University, Shanghai, 200032 China; 2 Key Laboratory of Breast Cancer in Shanghai, Fudan University Shanghai Cancer Center, Fudan University, Shanghai, 200032 China; 3 Department of Oncology, Shanghai Medical College, Fudan University, Shanghai, 200032 China

**Keywords:** Breast cancer, Prognostic markers

## Abstract

Triple-negative breast cancer (TNBC) is a great detriment to women’s health due to the lack of effective therapeutic targets. In this study, we employed an integrated genetic screen to identify a pivotal oncogenic factor, heterogeneous nuclear ribonucleoprotein U (HNRNPU), which is required for the progression of TNBC. We elucidated the pro-oncogenic role of HNRNPU, which can induce the proliferation and migration of TNBC cells via its association with DEAD box helicase 5 (DDX5) protein. Elevated levels of the HNRNPU-DDX5 complex prohibited the intron retention of minichromosome maintenance protein 10 (MCM10) pre-mRNA, decreased nonsense-mediated mRNA decay, and activated Wnt/β-catenin signalling; on the other hand, HNRNPU-DDX5 is located in the transcriptional start sites (TSS) of LIM domain only protein 4 (LMO4) and its upregulation promoted the transcription of LMO4, consequently activating PI3K-Akt-mTOR signalling. Our data highlight the synergetic effects of HNRNPU in RNA transcription and splicing in regulating cancer progression and suggest that HNRNPU may act as a potential molecular target in the treatment of TNBC.

## Introduction

Breast cancer is one of the most common malignant tumours in women [1]. Triple-negative breast cancer (TNBC) accounts for 15–20% of all invasive breast cancers [2–4]. TNBC is negative for oestrogen receptor (ER), progesterone receptor (PR) and human epidermal growth factor receptor (HER-2); as a result, hormone therapy and drugs that target HER-2 are not efficient [5]. In addition, TNBC is characterised by high heterogeneity and is more prone to recurrence and metastasis than other types [6, 7]. Therefore, TNBC is a great detriment to women’s health due to the lack of effective therapeutic targets. It is crucial to reveal the underlying mechanisms of TNBC and to develop effective therapeutic targets.

Previous studies have shown that transcriptional and posttranscriptional regulation are vital steps in breast cancer progression [8, 9]. Alternative splicing (AS) is an important type of posttranscriptional regulation and is essential for pre-mRNA maturation, in which introns are removed and exons are connected together [10]. There are many abnormal AS events that are widely observed in tumour biological processes, such as epithelial-mesenchymal transition (EMT), apoptosis, cell cycle, proliferation, metabolism, stress, immune destruction signalling and invasion [11]. For example, the protein encoded by the gene FGFR2 is a member of the fibroblast growth factor receptor family and plays a vital role in regulating cell proliferation, differentiation, migration and apoptosis and regulating embryonic development. FGFRIIIb and FGFRIIIc are two common splicing isoforms [12, 13]. FGFRIIIc can promote EMT [14]. Abnormal amplification of this gene has been observed in lung cancer and breast cancer [15, 16]. The regulation of AS relies on splicing factors that combine with pre-RNA [17]. The abnormal function of splicing factors may be the molecular mechanism for the development of many diseases [18]. In addition, dysfunctional splicing factors can act as oncogenes or tumour suppressor genes in the progression of tumours [19].

The heterogeneous nuclear ribonucleoprotein (hnRNP) family members are RNA-binding proteins (RBPs) involved in the regulation of AS as trans-acting factors [20]. Sequence analysis of hnRNPs revealed that they have at least one RNA-binding region and one auxiliary region [21]. There are two common RNA-binding regions of hnRNPs: the RNP-conserved sequence-RNA-binding domain (RNP-CS-RBD) and the K-homology domain (KH domain). The common auxiliary area of hnRNPs is the RGG domain (the arginine/glycine-rich domain). It was first discovered in the HNRNPU protein as a recurring Arg-Gly-Gly sequence [22]. The RGG region mediates protein‒RNA interactions, thereby affecting the binding activity of RNA and protein [23]. In the process of pre-mRNA alternative splicing regulation, hnRNPs can directly antagonise the spliceosome’s recognition of pre-mRNA splicing sites or hinder other splicing factors from combining with splicing sites and splicing enhancers [24]. Studies have shown that hnRNP A1, H and K can bind part of the 5’c-Src N1 exon [25]. In vitro hnRNP A1 can inhibit the splicing of c-Src. In recent years, a large number of studies have found that hnRNPs are highly expressed at the protein level in most tumours [21]. It has been reported that hnRNP A2/B1 can be detected in lung cancers [26]. Most lung cancer cell lines highly express hnRNP A2/B1, and the expression of hnRNP A2/B1 is higher in the early stage of lung cancer than in the late stage [27]. Recently, some researchers found that hnRNP A2/B1 can also activate Snail to induce EMT, thereby promoting liver cancer metastasis [28]. In summary, hnRNP has good prospects as a tumour marker.

In this study, we first identified the critical role of HNRNPU in promoting the progression of TNBC. Remarkably, HNRNPU is upregulated in breast cancer, especially in TNBC, and the high expression of HNRNPU indicates a poor prognosis. Both in vitro and in vivo experiments demonstrated that HNRNPU can function as an oncogene to promote breast cancer cell proliferation and migration. Then, we explored the binding between HNRNPU and DDX5, which can act as both a splicing factor and a transcription factor. We proposed a pro-oncogenic model in which on the one hand, the HNRNPU-DDX complex activates the Wnt/β-Catenin pathway by modulating the intron retention (RI) rate of minichromosome maintenance protein 10 (MCM10, a protein required for DNA replication) [29, 30]; on the other hand, the HNRNPU-DDX complex is located near the promoter of LIM domain only protein 4 (LMO4, an oncogene that was reported to promote the invasion and proliferation of cancer cells) and activates transcription and the PI3K/Akt pathway. However, the underlying therapeutic strategies need further research.

## Results

### HNRNPU is upregulated in breast cancer, and its high expression is associated with a poor prognosis

**Fig. 1:**
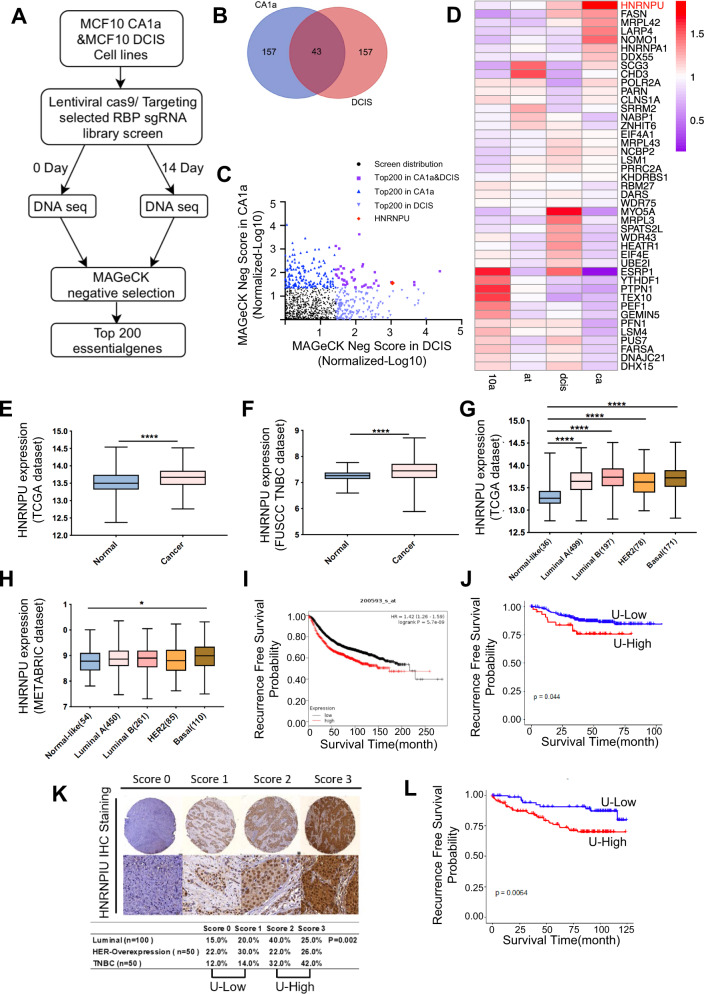
CRISPR/CAS9 screening library analysis revealed that HNRNPU is related to breast cancer progression. **A** Experimental outline of the CRISPR/CAS9 screening library. **B** Venn diagram of the top 200 genes in MCF10 CA1a and MCF10 DCIS cell lines based on negative screening. **C** Scatterplot of the top 200 candidate genes in our screening model cells. The top 200 genes are marked with different coloured dots. **D** Heatmap of the mRNA levels of the 43 overlapping candidate genes in MCF10A, MCF10 AT, MCF10 DCIS and MCF10 CA1a cells. **E** HNRNPU mRNA expression in breast cancer tissue and normal tissue in the TCGA database. **F** HNRNPU mRNA expression in breast cancer tissue and normal tissue in the FUSCC-TNBC database. **G** HNRNPU expression across the four molecular subtypes of breast cancer and normal tissue in the TCGA datasets. The number of patients is shown in brackets. Whiskers indicate the minimum and maximum values. **H** HNRNPU expression across the four molecular subtypes of breast cancer and normal tissue in the METABRIC datasets. The number of patients is shown in brackets. Whiskers indicate the minimum and maximum values. **I** Kaplan–Meier analysis of RFS using the Kaplan–Meier plotter database. **J** Kaplan–Meier analysis of RFS using the FUSCC-TNBC database. **K**, **L** Representative images of HNRNPU IHC staining in TNBC patients and statistical analysis of HNRNPU expression according to the IHC score. **L** Kaplan–Meier analysis of RFS in TNBC patients. A log-rank test was used to determine the statistical significance between the low HNRNPU expression group and the high HNRNPU expression group.

We recently developed a CRISPR/CAS9 screening library targeting the 1114 RNA-binding proteins (RBPs) to investigate the functional regulators in breast cancer (Fig. 1A). The virus produced after transfecting sgRNAs into HEK293T cells was used to infect MCF10 DCIS and MCF10 CA1a breast cancer cell lines. After puromycin selection, the cells were divided into two groups. One group was for Day 0 baseline cells; another group was transplanted into immunodeficient NOD-SCID mice, and the subcutaneous tumours were dissected after 14 days of cultivation. The DNA was extracted from the cells in the Day 0 and Day 14 groups for second-generation sequencing, and the MAGeCK algorithm was performed to negatively screen out functional genes. Taking the intersection of the top 200 genes in the negative screening, 43 potential genes were identified (Fig. 1B, C). The RNA-seq results of the MCF10 cell line progression model showed that HNRNPU transcriptome expression increased with the degree of tumour malignancy (Fig. 1D). Therefore, we chose HNRNPU as a potential essential gene for the following research.

Next, we further explored whether the expression of HNRNPU was clinically relevant to breast cancer patients. The Cancer Genome Atlas (TCGA) database and our single-centre database (Fudan University Shanghai Cancer Center database, FUSCC-database) showed that the mRNA expression of HNRNPU was upregulated in breast cancer tissues compared with noncancer tissues (Fig. 1A, E, F). The TCGA and METABRIC (Molecular Taxonomy of Breast Cancer International Consortium) databases demonstrated that HNRNPU was highly expressed in basal-like breast cancers (Fig. 1G, H). K–M plotter (http://kmplot.com/) indicated that patients with high HNRNPU expression had worse recurrence-free survival (RFS, Fig. 1I); the FUCSS-TNBC database displayed the same trend (Fig. 1J). The immunohistochemistry (IHC) staining analysis of the breast cancer tissue microarrays showed that the expression of HNRNPU was higher in TNBC than in other molecular subtypes (Fig. 1K). In addition, high expression of HNRNPU indicated a worse RFS (Fig. 1L).

### HNRNPU promotes cell proliferation and migration in vitro and vivo

**Fig. 2:**
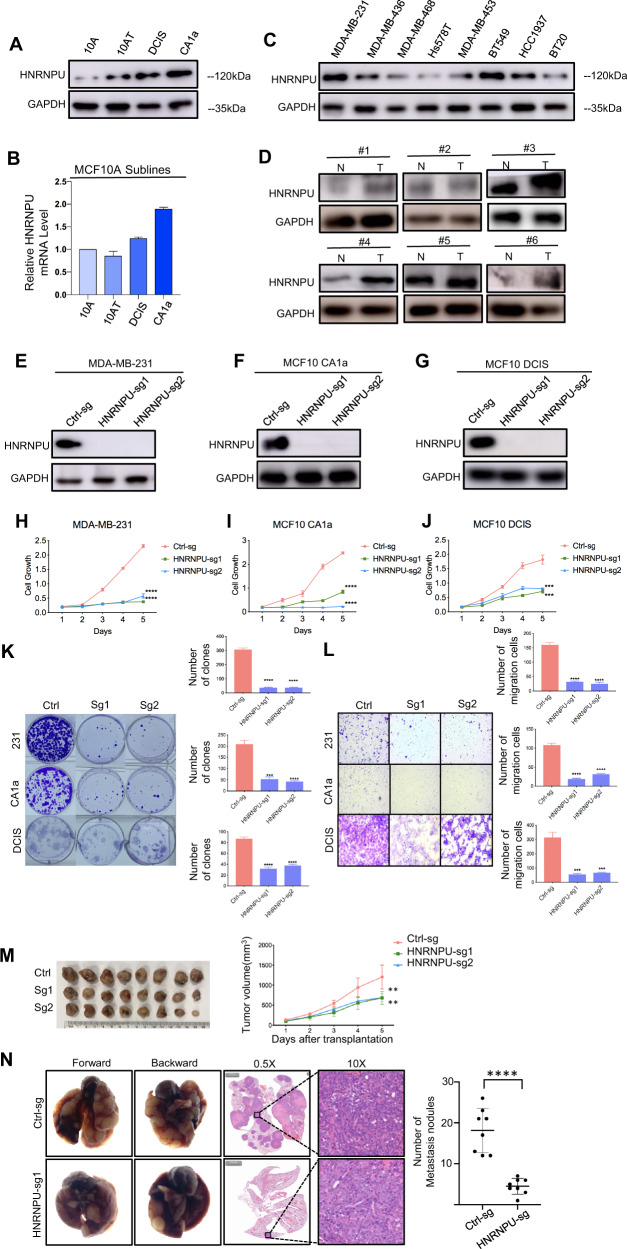
HNRNPU promotes TNBC cell proliferation and migration in vitro in vivo. **A** Western blot analyses of HNRNPU protein expression in MCF10 cells. **B** qPCR analyses of the HNRNPU mRNA levels in MCF10 cells. **C** Western blot analyses of HNRNPU protein expression in eight breast cancer cell lines. **D** Western blot analysis of HNRNPU protein expression in six pairs of matched breast cancer specimens and adjacent normal breast tissues. **E**–**G** Western blot analyses of CRISPR-mediated knockout of HNRNPU in MDA-MB-231, MCF10 CA1a and MCF10 DCIS cells. **H**–**J** Cell proliferation analyses using the CCK-8 assay in MDA-MB-231, MCF10 CA1a and MCF10 DCIS cells. **K** Cell proliferation analyses using a colony formation assay in MDA-MB-231, MCF10 CA1a and MCF10 DCIS cells. Representative images of surviving colonies (left) and corresponding quantitative results (right) are shown. **L** Transwell migration assay of MDA-MB-231, MCF10 CA1a and MCF10 DCIS cells. Representative images of cell migration (left) and quantitative results (right) are shown. **M** Photographs of harvested tumours (left) and tumour growth curves (right) are shown. **N** Representative photographs of metastatic lung nodules (left), H&E-stained sections of lung tissues (middle) and quantitative results of nodules (right) are shown. Data were presented as the mean ± SEM. ***p < 0.001, **p < 0.01, *p < 0.05, n.s. not significant.

To elucidate the tumorigenic role of HNRNPU, we first verified the protein and mRNA expression levels in the MCF10 cell line progression model (Fig. 2A, B). Consistent with the above RNA-sequencing data, HNRNPU expression increased with the level of tumour malignancy. We assessed the expression of HNRNPU in other breast cancer cell lines, and high expression was found in MDA-MB-231 cell lines (Figs. 2C and S1A). Therefore, we selected MDA-MB-231, MCF10 CA1a and MCF10 DCIS cells for HNRNPU knockout functional experiments. In addition, we examined HNRNPU expression using six pairs of tumour and adjacent normal tissues, and the results showed that HNRNPU levels were elevated in breast tumour tissues (Fig. 2D).

To determine the functional role in vitro, we knocked out HNRNPU through a CRISPR‒Cas9 approach (Fig. S1B). Figure 2E–G demonstrate the knockout efficiency in MDA-MB-231, MCF10 CA1a and MCF10 DCIS cells. CCK-8 tests and colony formation assays revealed that knocking out HNRNPU could significantly inhibit cell proliferation (Fig. 2H–K). Transwell migration assays showed that reduced HNRNPU dramatically weakened the cell migration capacity (Fig. 2J). To fulfil the function of HNRNPU in vivo, we injected HNRNPU knockout cells and control cells into the mammary fat pads of 6-week-old female NOD/SCID mice. After 28 days of cultivation, tumours were dissected and analysed, and the results showed that tumours in the HNRNPU KO group were significantly smaller than those in the control group (Fig. 2M). In addition, HNRNPU knockout cells and control cells were injected into the lateral tail vein of 6-week-old female NOD/SCID mice. After 6 weeks of injection, knocking out HNRNPU dramatically decreased the number of metastatic tumours in the lungs of mice (Fig. 2N).

Above all, these data demonstrated that HNRNPU promotes cell proliferation and migration in vitro and vivo.

### HNRNPU binds to DDX5 to form a complex to exert tumorigenic functions

**Fig. 3:**
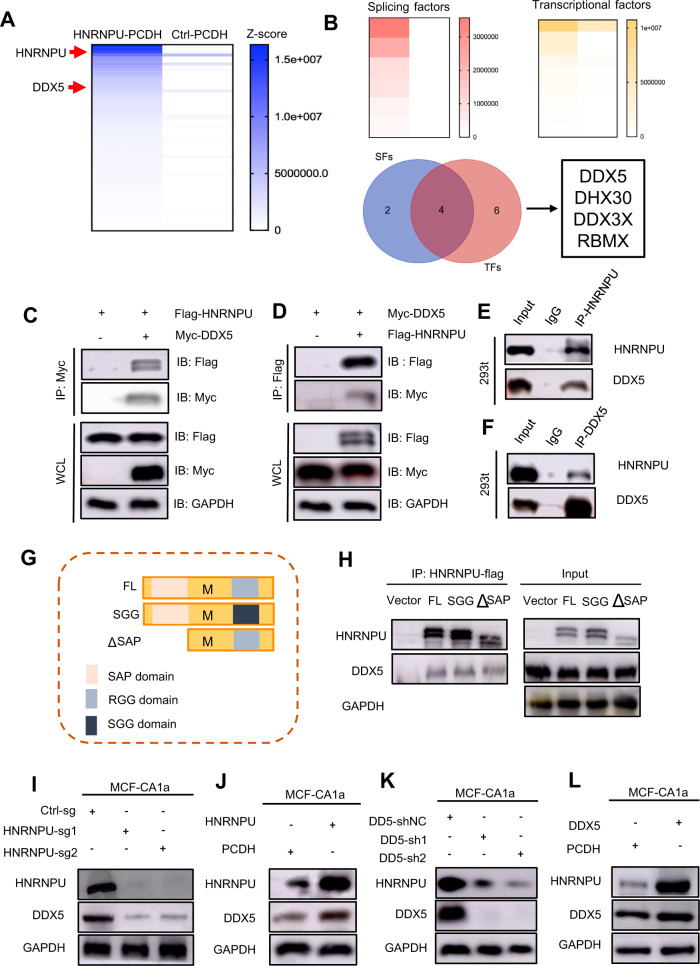
HNRNPU interacts with DDX5. **A** Heatmap of proteins identified from Flag-HNRNPU IP/MS analysis. **B** Heatmap of splicing factors and transcription factors identified from Flag-HNRNPU IP/MS analysis, Venn diagram of splicing factors and transcription factors identified from Flag-HNRNPU IP/MS analysis. **C**, **D** Coimmunoprecipitation analysis shows the interaction between HNRNPU and DDX5. **E**, **F** Coimmunoprecipitation analysis shows the interaction between endogenous HNRNPU and DDX5 in HEK293T cells. **G** Schematic diagram of HNRNPU domains and constructions of two HNRNPU mutants. All mutants were FLAG-tagged. **H** Coimmunoprecipitation analysis shows the interaction between endogenous Flag-tagged HNRNPU truncation mutants and DDX5 in HEK293T cells. **I**, **J** Effects of HNRNPU expression on DDX5 protein levels in MCF10 CA1a cells by immunoblotting analysis. **K**, **L** Effects of DDX5 expression on HNRNPU protein levels in MCF10 CA1a cells by immunoblotting analysis.

To address how HNRNPU performs its biological functions, we sought to identify potential interacting proteins. 3XFlag-tagged overexpression HNRNPU protein (HNRNPU-Flag) was immunoprecipitated with anti-Flag antibody from HEK293T cells, and then we conducted protein immunoprecipitation mass spectrometry (IP-MS). The pulled-down proteins are displayed in Fig. 3A. Interestingly, six of these proteins were splicing factors, and ten of these proteins were transcription factors. By intersecting these proteins, we obtained four proteins with both splicing and transcription functions. We focused our sights on the top-ranked DDX5 (Fig. 3B). DDX5 (DEAD (Asp‐Glu‐Ala‐Asp) box polypeptide 5), also known as RNA helicase p68, is involved in a variety of cellular processes, including transcription, alternative splicing, translation and precursor messenger RNA processing [31]. In addition, DDX5 was reported to be abnormally expressed in a variety of tumours and to play a crucial role in tumour progression [32]. For example, DDX5 acts as a transcriptional coactivator of many oncogenic transcription factors, including nuclear factor-κβ (NF-κβ), oestrogen receptor α (ERα), β-catenin, and androgen receptor [33].

To validate the interaction between HNRNPU and DDX5, coimmunoprecipitation (Co-IP) experiments were conducted between endogenous HNRNPU and DDX5-Myc as well as between endogenous DDX5 and HNRNPU-Flag in HEK293T cells (Fig. 3C, D). Endogenous Co-IP further revealed the combination (Fig. 3E). Immunofluorescence (IF) experiments demonstrated that HNRNPU and DDX5 were colocalized in the nucleus (Fig. S2A). The SAP domain, which is located in the N-terminal of HNRNPU, binds to DNA; the RGG domain, located in the C-terminal, is an RNA-binding domain [34]. After deleting the SAP domain or mutating the RGG domain into a non-functional SGG domain, the interaction between HNRNPU and DDX5 remained unaffected, which may indicate that the binding of the two proteins is independent of the SAP/RGG domain (Fig. 3G, H). Therefore, we suppose that the binding of HNRNPU and DDX5 is DNA/RNA-independent. Then, we knocked out and overexpressed HNRNPU, and the DDX5 protein level was correspondingly reduced or increased (Fig. 3I, J). However, the mRNA level of DDX5 remained unchanged (Fig. S2B). Conversely, the knockdown and overexpression of DDX5 resulted in similar effects (Figs. 3K, L and S2C). Based on these findings, we propose that HNRNPU and DDX5 form a complex to exert tumorigenic functions.

### HNRNPU modulates alternative splicing events by interacting with DDX5

**Fig. 4:**
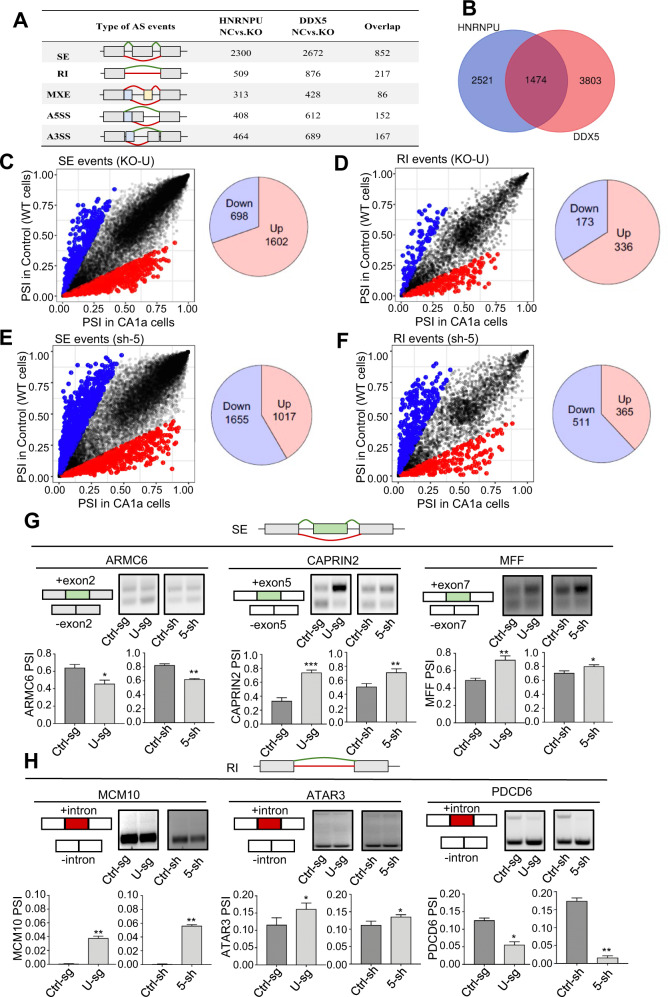

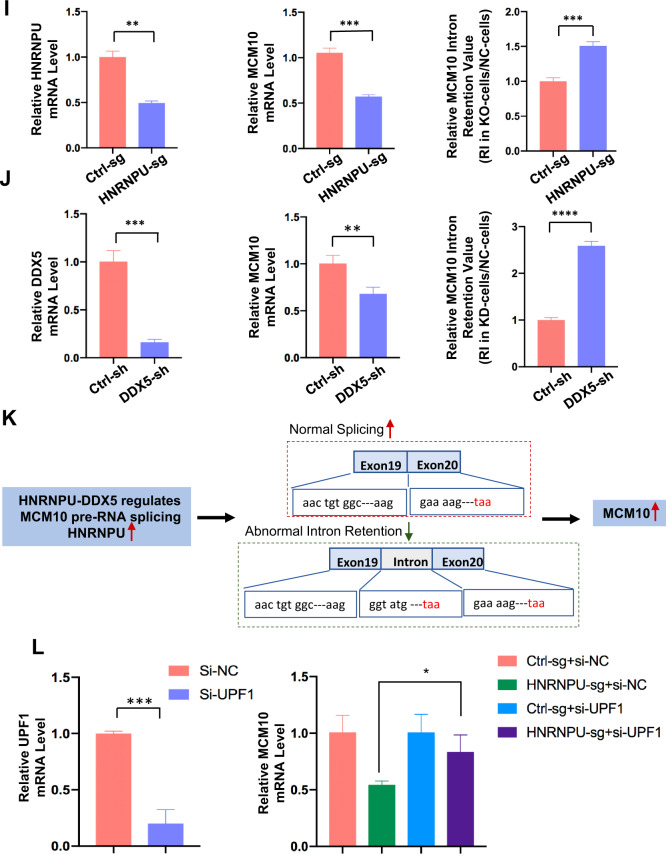
HNRNPU modulates alternative splicing events by interacting with DDX5. **A** Quantification of AS events after HNRNPU was knocked out or DDX5 was knocked down. **B** Venn diagram of the AS events regulated by HNRNPU and DDX5. **C**, **D** PSI (Percent spliced in) profiles of SE and RI events identified in control and HNRNPU knockout cells (left). The coloured dots represent significantly upregulated (red) or downregulated (blue) events in HNRNPU knockout cells compared with control cells (left). Pie chart shows the quantification of upregulated (pink) or downregulated (purple) SE and RI events after HNRNPU was knocked out (right). **E**, **F** PSI profiles of SE and RI events identified in control and DDX5 knockdown cells (left). The coloured dots represent significantly upregulated (red) or downregulated (blue) events in DDX5 knockdown cells compared with control cells (left). Pie chart shows the quantification of upregulated (pink) or downregulated (purple) SE and RI events after DDX5 was knocked down (right). **G** Representative RT‒PCR validation of HNRNPU-regulated SE events and DDX5-regulated SE events. The structure of each isoform is illustrated in the diagrams. Individual data points are presented (*n* = 3; ***p < 0.001, **p < 0.01, *p < 0.05; Student’s *t*-test). **H** Representative RT‒PCR validation of HNRNPU-regulated RI events and DDX5-regulated RI events. The structure of each isoform is illustrated in the diagrams. Individual data points are presented (*n* = 3; ***p < 0.001, **p < 0.01, *p < 0.05; Student’s *t*-test). **I** HNRNPU knockout increases the MCM10 splicing for intron retention and decreases MCM10 mRNA expression. Quantitative real-time PCR analysis of MCM10 mRNA expression and intron retention value. **J** DDX5 knockdown increases the MCM10 splicing for intron retention and decreases MCM10 mRNA expression. Quantitative real-time PCR analysis of MCM10 mRNA expression and intron retention value. **K** Schematics of the MCM10 alternative splicing pattern regulated by HNRNPU and DDX5. The first stop codon in the intron is indicated with red letters. **L** HNRNPU knockout and control MCF10 CA1a cells were transfected with control siRNA and UPF1 siRNA. MCM10 mRNA expression was examined by Quantitative real-time PCR.

To gain further insight into the tumorigenesis mechanisms, we conducted high-throughput mRNA sequencing (RNA-seq) using HNRNPU knockout and DDX5 knockdown MCF10 CA1a and MCF10 DCIS cells. There were a total of 3995 HNRNPU-regulated and 5277 DDX5-regulated AS events in MCF10 CA1a cells. Different types of AS events are shown in Fig. 4A, and they were skipped exons (SE), retained introns (RI), mutually exclusive exons (MXE), alternative 5′ ss exons (A5SSs) and alternative 3′ ss exons (A3SSs) (Fig. S3). Notably, approximately one-third of HNRNPU-regulated AS events and DDX5-regulated AS events overlapped, which may indicate that HNRNPU and DDX5 are coordinated to regulate AS (Fig. 4B). Then, SE and RI, the two most common types of regulated AS events, were analysed. Knocking out HNRNPU resulted in 1602 upregulated SE events, 698 downregulated SE events, 336 upregulated RI events and 173 downregulated RI events (Fig. 4C, D), while knocking down DDX5 led to 1017 upregulated SE events, 1655 downregulated SE events, 365 upregulated RI events and 511 downregulated RI events (Fig. 4E, F). The splicing analyses in MCF10 DCIS cell lines showed the same trend (Fig. S4A–F). Three SE events and three RI events were verified to be consistent with RNA-seq analyses by semiquantitative RT‒PCR assays (Fig. 4G, H).

QPCR was further used for validation, which was consistent with our RNA-sequencing data showing that the intron retention of MCM10 increased and the mRNA level of MCM10 decreased after HNRNPU knockout or DDX5 knockdown (Fig. 4I, J). Nonsense-mediated decay (NMD) is a quality control monitoring mechanism for the degradation of abnormal mRNA [35]. Intron-retention mRNA transcripts contain early termination codons, which could destroy the open reading frame and trigger NMD degradation [35]. The mRNA analysis of MCM10 showed that the retained intron contained the stop codon of TAA in the transcript (Fig. 4K). To further demonstrate whether the NMD pathway was involved in the regulation of MCM10 transcripts induced by HNRNPU, we downregulated the key factor UPF1 of NMD with siRNA in MCF10 CA1a cells. qRT-PCR was used to evaluate MCM10 expression after UPF1 knockdown, and the results showed that suppression of UPF1 resulted in a significant increase in MCM10 mRNA compared with control siRNA-treated HNRNPU knockdown (Figs. 4L and S4O).

### HNRNPU regulates transcription by interacting with DDX5

**Fig. 5:**
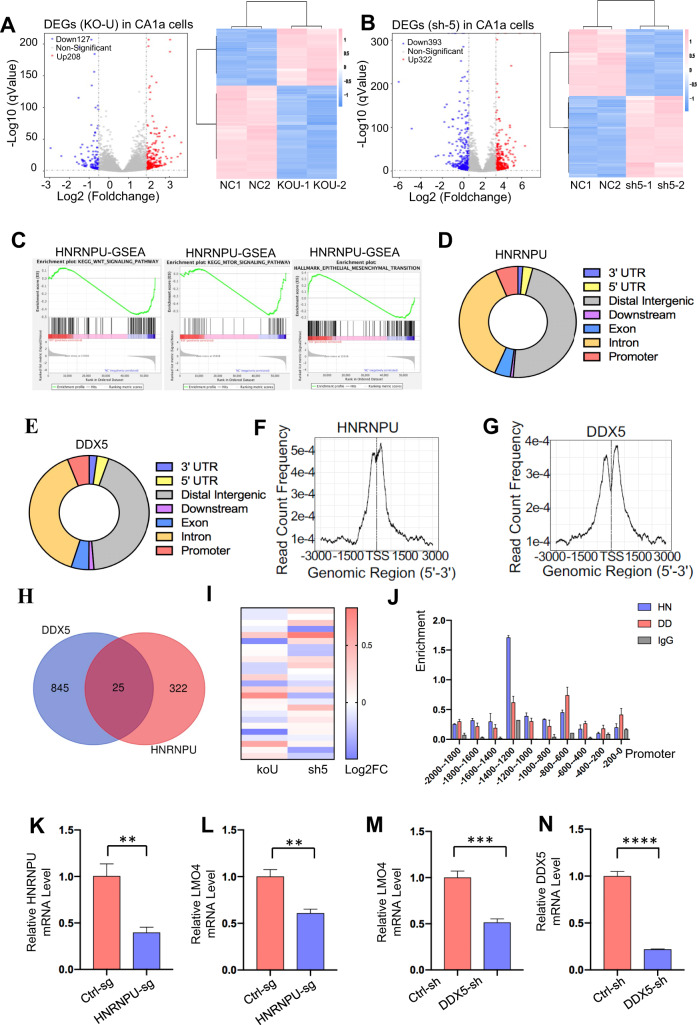
HNRNPU regulates transcription by interacting with DDX5. **A** Volcano plot of differentially expressed genes after the knockout of HNRNPU (left). Red and blue indicate high and low expression, respectively. Heatmap representing the unsupervised hierarchical clustering of mRNA expression levels in HNRNPU knockout and control MCF10 CA1a cells (right). **B** Volcano plot of differentially expressed genes after the knockdown of DDX5 (left). Red and blue indicate high and low expression, respectively. Heatmap representing the unsupervised hierarchical clustering of mRNA expression levels in DDX5 knockdown and control MCF10 CA1a cells (right). **C** GSEA results were plotted to visualise the correlation between the expression of HNRNPU and related carcinogenic pathways in MCF10 CA1a cells. **D** Pie chart shows the genomic distribution of CHIP-Seq peaks for HNRNPU. 5**E** Average genome-wide occupancies of HNRNPU along the transcription unit. The gene body length was aligned to 3000 bp upstream and downstream of the TSS. **F** Pie chart shows the genomic distribution of CHIP-Seq peaks for DDX5. **G** Average genome-wide occupancies of DDX5 along the transcription unit. The gene body length was aligned to 3000 bp upstream and downstream of the TSS. **H** Venn diagram illustrating the overlap of HNRNPU- and DDX5-occupied genes. **I** Heatmap representing the mRNA expression levels of 25 overlapping genes (in Fig. 5H) in HNRNPU knockout and DDX5 knockdown cells. **J** HNRNPU and DDX5 enrichment on the Lmo4 promoter was analysed via ChIP‒qPCR analysis. **K**, **L** HNRNPU knockout decreases LMO4 mRNA expression. Quantitative real-time PCR analysis of HNRNPU and LMO4 mRNA expression. **M**, **N** DDX5 knockdown decreases LMO4 mRNA expression. Quantitative real-time PCR analysis of DDX5 and LMO4 mRNA expression.

In addition to AS analysis, transcription profiling revealed the differentially expressed genes. After knocking out HNRNPU, 208 genes were upregulated, while 127 genes were downregulated in MCF10 CA1a cell lines (Fig. 5A). Knocking down DDX5 caused the upregulation of 322 genes and the downregulation of 393 genes (Fig. 5B). The differentially expressed genes in control versus DDX5 knockdown MCF10 DCIS cell lines are shown in Figure S4G, H. Gene set enrichment analysis (GSEA) revealed that HNRNPU could significantly regulate the Wnt signalling pathway, mTOR signalling pathway and EMT (Figs. 5C and S4M, N), and DDX5 significantly regulated EMT and myc targets (Fig. S4J–L). A gene ontology (GO) analysis was performed to demonstrate that the HNRNPU and DDX5 overlapping downstream genes were involved in poly (A) RNA-binding, DNA repair, cell division and cell‒cell adhesion (Fig. S4I).

To investigate how HNRNPU and DDX5 regulate transcription, ChIP-seq assays were performed. Genomic distribution analysis showed that the top three identified regions of HNRNPU and DDX5 were distal intergenic regions, intron regions, and promoter regions (Fig. 5D, E). Promoter regions include the transcriptional start sites (TSS) and can extend for ~35–40 bp upstream and/or downstream of the TSS [36], and promoters mainly function by regulating the initiation of transcription [37]. Therefore, we focused our attention on promoter regions. Figure 5F, G further demonstrated that HNRNPU and DDX5 were enriched in the TSS. There were 25 genes after overlapping HNRNPU and DDX5 identified binding regions corresponding to within 2 kb of promoters (Fig. 5H, I). LMO4 is an oncogene and was reported to promote the invasion and proliferation of gastric cancer by activating PI3K/Akt/mTOR signalling [38]. ChIP-qPCR revealed that HNRNPU and DDX5 were mainly enriched in the −1400 to −1200 and −800 to −600 regions downstream of the TSS of LMO4 (Fig. 5J). QPCR showed that the LMO4 mRNA level decreased with the downregulation of HNRNPU and DDX5 (Fig. 5K–N). HNRNPU interacts with DDX5 to regulate LMO4 transcription through enrichment near its TSS.

### HNRNPU activates the Wnt/β-Catenin pathway and PI3K/Akt/mTOR pathway by regulating MCM10 and LMO4

**Fig. 6:**
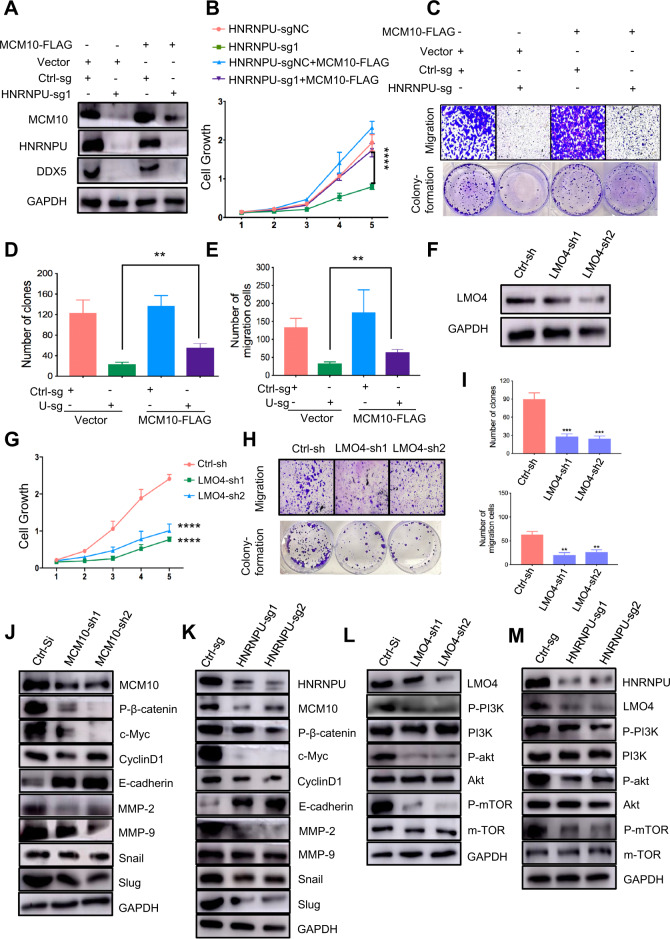
HNRNPU activates the Wnt/β-Catenin pathway and PI3K/Akt/mTOR pathway by regulating MCM10 and LMO4. **A** Western blot analysis of endogenous HNRNPU knockout MCF10 CA1a cells stably expressing exogenous MCM10-FLAG. **B** Cell proliferation assays of the cells described in Fig. 6A. **C**–**E** Colony formation assays and Transwell assays of the cells described in Fig. 6A. Representative images (**C**) and quantitative results (**D**, **E**) are shown. **F** MCF10 CA1a cells were transfected with two different shRNAs targeting LMO4 (LMO4-sh1, LMO4-sh2) or negative control shRNA (Ctrl-sh). Cells were analysed by immunoblotting after 48 h of transfection. **G** Cell proliferation assays of the cells described in Fig. 6F. **H**, **I** Colony formation assays and Transwell assays of the cells described in Fig. 6F. Representative images (**H**) and quantitative results (**I**) are shown. Data were presented as the mean ± SEM. ***p < 0.001, **p < 0.01, *p < 0.05, n.s. not significant. **J** Western blot analysis showing the levels of P-β-catenin, c-Myc, CyclinD1, E-cadherin, MMP-2, MMP-9, Snail and Slug when the expression of MCM10 was decreased. **K** Western blot analysis showing the levels of MCM10, P-β-catenin, c-Myc, CyclinD1, E-cadherin, MMP-2, MMP-9, Snail and Slug when the expression of HNRNPU was decreased. **L** Western blot analysis shows the levels of P-PI3K, PI3K, P-akt, Akt, P-mTOR and mTOR when the expression of LMO4 was decreased. **M** Western blot analysis shows the levels of LMO4, P-PI3K, PI3K, P-akt, Akt, P-mTOR and mTOR when the expression of HNRNPU was decreased.

Given that HNRNPU regulates MCM10, we attempted to verify MCM10 as a potential regulator of TNBC progression downstream of HNRNPU. To confirm this, HNRNPU knockout and DDX5 knockdown MCF10 CA1a cells were transfected with MCM10-FLAG plasmid or vector (Figs. 6A and S5A). Cell growth and colony formation assays indicated that overexpression of MCM10 significantly attenuated the decrease in the proliferation capacity of HNRNPU knockout and DDX5 knockdown cells (Fig. 6B–D and S5B–D). Moreover, the Transwell migration assays revealed that the overexpression of MCM10 markedly attenuated the decrease in migratory capacity induced by HNRNPU knockout or DDX5 knockdown (Figs. 6C–E and S5B, E). Together, these results showed that the suppression of cell proliferation and tumorigenesis by HNRNPU knockdown could be attenuated by overexpression of MCM10, indicating that HNRNPU promotes TNBC, at least partially, through its regulation of MCM10.

A previous study showed that MCM10 facilitates the migration potential of breast cancer cells via Wnt/β‐catenin signalling and is positively correlated with a poor prognosis [39]. To verify this mechanism, we knocked down MCM10 in CA1a cell lines. The representative Wnt/β‐catenin signalling proteins phospho-β-catenin and C-myc were downregulated, and the downstream molecules involved in EMT, including MMP-2, MMP-9, snail and slug, were also downregulated (Fig. 6J). GSEA demonstrated that HNRNPU and DDX5 alters the expression of EMT-related factors at the mRNA level (Figs. 5C and S4J). Figures 6K and S5F verified that knocking out HNRNPU and knocking down DDX5 suppressed the Wnt/β-Catenin pathway and inhibited EMT at the protein level.

In addition, it was reported that LMO4 promoted the invasion and proliferation of cancer by activating the PI3K/Akt/mTOR signalling pathway [38]. To gain insights into the function LMO4 exerted in breast cancer, knockdown LMO4 stable MCF10 CA1a cell lines were constructed (Fig. 6F). CCK-8 and colony formation assays revealed that the knockdown of LMO4 could significantly inhibit cell proliferation (Fig. 6G–I). Transwell migration assays showed that reduced LMO4 expression dramatically weakened the cell migration capacity (Fig. 6H, I). Moreover, Western blotting revealed that LMO4 knockdown caused a decrease in the PI3K/Akt/mTOR pathway (Fig. 6L). GSEA demonstrated that HNRNPU and DDX5 modulate the mTOR pathway transcriptionally (Figs. 5C and S4L). Western blotting confirmed the downregulation of PI3K/Akt/mTOR signalling induced by HNRNPU and DDX5 (Figs. 6M and S5G).

Taken together, these results indicate that HNRNPU activates the Wnt/β-Catenin pathway and PI3K/Akt/mTOR pathway to promote TNBC progression.

### The HNRNPU-DDX5 complex results in a poor prognosis for breast cancer patients and the underlying molecular mechanism was clarified

**Fig. 7:**
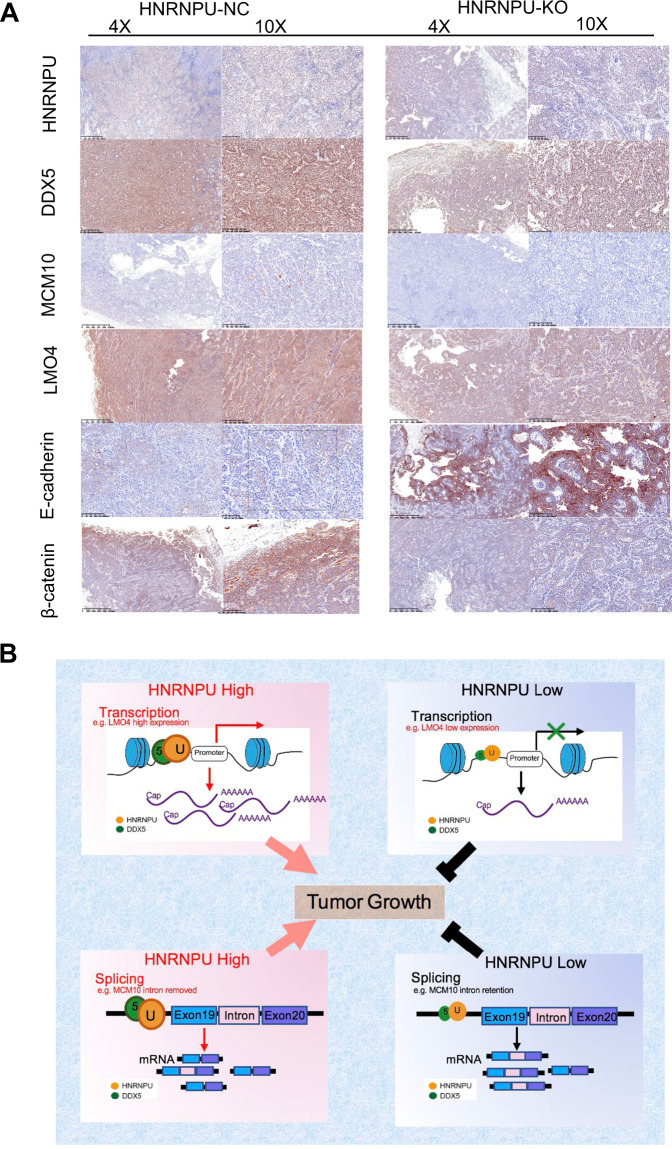
The HNRNPU-DDX5 complex is related to poor breast cancer prognosis, and the underlying molecular mechanism model was clarified. **A** Representative images of immunohistochemical staining as indicated in the xenografted tumours. **B** The proposed working model of this study.

As determined by IHC staining analysis, knocking down HNRNPU resulted in a decrease in MCM10 expression, a decrease in LMO4 expression and an increase in E-cadherin expression, reflecting EMT inhibition (Fig. 7A). To investigate whether the HNRNPU downstream genes were associated with clinical outcome, K–M plotter analysis was performed, showing that high expression of MCM10 and LMO4 was significantly related to worse overall survival (OS) and RFS (Fig. S6A–D). Then, we evaluated the correlation between HNRNPU and MCM10 and LMO4, and a strong positive correlation was found (Fig. S6E, F). These results supported the hypothesis that HNRNPU promotes the progression of TNBC through the regulation of MCM10 and LMO4.

The model shown in Fig. 7B illustrates how HNRNPU promotes TNBC progression. In cancer cells with high HNRNPU expression, on the one hand, HNRNPU localises near the promoter of LMO4 and promotes PI3K/Akt/mTOR signalling by activating transcription; on the other hand, HNRNPU regulates the alternative splicing of MCM10, suppresses nonsense-mediated mRNA decay (NMD) caused by intron retention and activates the Wnt/β-catenin pathway. Overall, HNRNPU, as an oncogene, epigenetically modulates the progression of TNBC.

## Discussion

In this study, we screened the RNA-binding protein HNRNPU, which may be tumorigenic, through the CRISPR/CAS9 library. Existing studies on HNRNPU-involved tumours have mainly focused on the proliferative properties in cervical cancer [40], hepatocellular carcinoma [41] and pancreatic ductal adenocarcinoma [42]. However, the function and tumorigenic role of HNRNPU in breast cancer have rarely been reported, so this finding attracted our interest. Our investigation showed that HNRNPU could promote the proliferation and migration of breast cancer cells in vitro and vivo, and this has been verified in patient data showing that the expression of HNRNPU in cancer tissues was higher than that in adjacent tissues. Moreover, HNRNPU was significantly associated with poor RFS. These results were consistent with the results for HNRNPU in the majority of human cancers. Therefore, our research showed that HNRNPU serves as an oncogene in breast cancer.

HNRNPU has been biochemically characterised as an RNA-binding protein, and a previous study demonstrated that HNRNPU functions as a global splicing regulator [43]. Xiao et al. identified thousands of direct HNRNPU binding targets in the human genome, deduced its GU-rich binding consensus, and provided a series of lines of evidence suggesting that HNRNPU may regulate alternative splicing mainly by modulating U2 snRNP maturation [43]. Our Co-IP assays revealed that HNRNPU and DDX5 bind to one another, and we proposed a new model in which HNRNPU regulates AS and transcription. RNA-seq and CHIP-seq analyses suggested that HNRNPU modulates alternative splicing and transcription by interacting with DDX5. The RNA helicase DDX5 is a member of a large family of highly conserved proteins that are involved in gene expression regulation [44]. Dardenne et al. proposed that the RNA helicase activity of DDX5 and DDX17 favours the binding of HNRNP H/F to G-tracts that can form G-quadruplex structures and assist them in their splicing-enhancer function [44]. Yeon J et al. reported a substantial overlap of AS events regulated by DDX5 and HNRNP A1 and elucidated that HNRNP A1 and DDX5 coregulate AS [45]. Although in our study we innovatively discovered that HNRNPU and DDX5 interact to co-ordinately regulate transcription and splicing, the possibility that HNRNPU may regulate alternative splicing through various other mechanisms, such as binding to other pre-RNAs or interact with other splicing factors, is still not excluded.

Aberrant AS events frequently occur in tumours [17–19]. The role of HNRNPU in alternative splicing regulation was unclear in breast cancer until now. In this study, we analysed RNA-seq data in HNRNPU knockout and DDX5 knockdown breast cancer cells. The results showed vast overlaps of downstream AS events regulated by HNRNPU and DDX5. This was consistent with our supposition that HNRNPU and DDX5 had synergistic cancer‐promoting effects. We focused our attention on MCM10, a protein that is required for DNA replication [29, 30]. Our study showed that HNRNPU inhibition increased the RI of MCM10 between exons 19 and 20 and decreased MCM10 expression by activating the NMD pathway. The limitation of this study is that the observed RI rate is small. Maybe we only elucidated one of the mechanisms by which HNRNPU regulates MCM10 expression, and we leave open the possibility that HNRNPU may contribute to regulate MCM10 through multiple other mechanisms. Wei‐Dong *Yan* et al. reported that MCM10 activated Wnt/β‐catenin signalling to promote breast cancer metastasis in MDA-MB-231 cells [39], and we further validated this in MCF10 CA1a cells. In addition, the clinical data and IHC staining analysis demonstrated that MCM10 was associated with migration ability and indicated poor breast survival. Taken together, these findings may elucidate how HNRNPU promotes TNBC progression via AS mechanisms.

LMO4, a LIM-only transcriptional regulator, was reported to induce breast cancer invasion and predict poor outcomes [46, 47]. Benyu Liu et al reported the mechanism underlying the activation of the pro-oncogenic protein LMO4: Yeats4 recruits the Dot1l–RNA Pol II complex to the LMO4 promoter in innate lymphoid cells [48]. Our CHIP-seq and CHIP-qPCR data showed a similar pattern in breast cancer cells.: HNRNPU and DDX5 are enriched in the TSS of LMO4 and then activate LMO4 transcription. These results explained the mechanism by which LMO4 expression is activated. Regarding how LMO4 functions when it is upregulated, Ning Wang et al reported that LMO4 promoted the invasion and proliferation of gastric cancer by activating PI3K-Akt-mTOR signalling [38]. Similarly, our data showed that LMO4 can promote the phosphorylation of PI3K and activate its downstream signalling pathway in TNBC. In addition, LMO4 promotes the invasion of TNBC. Accordingly, LMO4 is a predictor of poor outcomes in breast cancer.

Overall, our study innovatively elucidated that HNRNPU induces the proliferation and migration of TNBC cells in vivo and in vitro and that HNRNPU is associated with a poor prognosis in breast cancer patients. Our results further emphasise the important epigenetic regulation mechanism of the HNRNPU-DDX5 complex. On the one hand, HNRNPU upregulation promotes the RI of MCM10, inhibits nonsense-mediated mRNA decay, and activates Wnt/β‐catenin signalling; on the other hand, HNRNPU is located in the LMO4 TSS region, its upregulation promotes the transcription of LMO4 and finally activates PI3K-Akt-mTOR signalling. Our study proposed a novel perspective regarding HNRNPU and the underlying pro-oncogenic mechanisms. Notably, HNRNPU can act as a potential molecular target, and a molecular basis was also provided for the treatment of TNBC.

Our study is the first to propose that HNRNPU and DDX5 interact to co-ordinately regulate transcription and splicing. However, the underlying mechanisms by which HNRNPU and DDX5 regulate splicing and transcription and the timeline by which they trigger transcriptional regulation and regulate splicing need to be further explored. Further research on how to use HNRNPU in clinical therapy is also needed.

## Materials and methods

### Cell cultures and reagents

MCF10A, MDA-MB-231, MDA-MB-436, MDA-MB-468, Hs578t, HCC1937 and the human embryonic kidney HEK293T cell lines were obtained from the Shanghai Cell Bank Type Culture Collection Committee (CBTCCC) in 2012. MCF10 AT, MCF10 DCIS, MCF10 CA1a, MDA-MB-453, BT549 and BT20 cell lines were kindly provided by Prof. Guo-Hong Hu (Shanghai Institutes for Biological Sciences, Shanghai, China) in 2014. The MCF10 cell lines were maintained in DMEM:F12 medium (Gibco) with 5% horse serum (Gibco), 10 mg/mL insulin (Sigma), 20 ng/mL EGF (Invitrogen), 0.5 mg/mL hydrocortisone (Sigma), 100 ng/mL cholera toxin (Sigma) and 1% penicillin‒streptomycin (BasalMedia). MDA-MB-231 and HEK293T cell lines were cultured in DMEM (BasalMedia) with 10% foetal bovine serum (Gibco) and 1% penicillin‒streptomycin (BasalMedia). All of the above cells were grown in a humidified environment consisting of 95% air and 5% CO2 at 37 °C and were authenticated by short tandem repeat profiling (STR). All the cell lines were cultured according to standard protocols, and the cells were not passaged more than six times from collection to use.

### Plasmid transfection and viral infection

The CRISPR/CAS9 system was used to construct HNRNPU knock-out stable cell lines. The sgRNA of HNRNPU was designed according to the Web-based CRISPR design tool from the Zhang lab (http://www.genome-engineering.org/) (Table. S1). The lenti-Cas9-Blast (Addgene, USA, #52962) and lentiguide-Puro (Addgene, #52963) vectors were provided by the Feng Zhang laboratory. First, HEK293T cells were transfected with lenti-Cas9-Blast plasmid using polyethyleneimine (PEI). The produced virus solution was collected after 48 h. Then, MCF10 cell lines were infected with the cas9 virus solution, screened by blasticidin (Invitrogen) and validated by western blotting. Second, six sgRNAs targeting HNRNPU were cloned into the lentiGuide-Puro vectors using BsmBI enzyme (NEB, #R0739) and T4-DNA Ligase (NEB, #M0202S) and validated by Sanger sequencing. Single sgRNA virus was generated by the transfection of HEK293 cells. After harvest, the six single sgRNA viruses were introduced into Cas9 cells. Forty-eight hours after infection, the stably integrated cells were selected with 1–2 mg/mL puromycin for 7 days. Western blotting experiments showed the knockout efficiency of six sgRNAs (Fig. S1B) and sgRNA1 and sgRNA2 were used for further study.

Stable DDX5, LMO4 and MCM10 knockdown cell lines were constructed by shRNA, and the shRNA sequence is shown in Table S1. Virus packaging, infection and verification methods were the same as above.

### Western blotting assays

All the primary antibodies applied are shown in Table S2. There were four main steps in Western blotting. First, protein extraction was performed. Cells or tissues were lysed with RIPA extraction buffer (Thermo, 89900) containing protease and phosphatase inhibitor cocktails, and the supernatant solution was quantified after centrifugation. Second, SDS‒PAGE separated proteins through electrophoresis, and then the protein was transferred to PVDF membranes (Millipore, IPVH00010, ISEQ00010). Third, the PVDF membranes were blocked with 5% bovine serum albumin (BSA) and incubated with antibodies according to the instructions. Finally, the bands were tested and analysed by ImageJ.

### Immunoprecipitation mass spectrometry and immunoprecipitation assays

HEK293T cells were transfected with HNRNPU-flag plasmid to overexpress HNRNPU exogenously. Then, the 293T cells were lysed in NP40 buffer (0.5% NP40, 50 mM Tris-HCl, pH 8, 150 mM NaCl, 10% glycerol, 2 mM MgCl2 and 1 mM EDTA) supplemented with protease inhibitor cocktail, and the supernatant was incubated with antibody overnight at 4 °C. Protein G magnetic beads were added for 2–4 h of incubation at 4 °C, and the protein-bead mix was washed with NP40 buffer three times followed by boiling in 1x SDS loading buffer. After SDS‒PAGE electrophoresis, the entire lane was cut for sequencing by mass spectrometry analysis (Novogene Bioinformatics Institute, Beijing, China). Protein identification was analysed by Proteome Discoverer 2.2 (PD2.2, Thermo) in the human RefSeq protein database (National Center for Biotechnology Information). In addition, after SDS‒PAGE, the proteins were subjected to immunoblotting analysis.

### Immunofluorescence assays

Adherent MCF10 CA1a cells were washed in PBS, fixed in 4% paraformaldehyde, permeabilized in 0.1% Triton X-100, and blocked in 1% BSA. The cells were incubated with antibodies in 1% BSA according to the instructions. Gold Antifade Mountant with DAPI (Invitrogen, USA, P36931) was used for DNA staining. LeicaSP5 confocal laser scanning microscopy (Leica Microsystems, Buffalo Grove, USA) was used for immunofluorescence imaging analyses.

### RNA-seq analysis

HNRNPU knockout and DDX5 knockdown cells, as well as normal controls, were collected, and total RNA was extracted using TRIzol Reagent (Invitrogen) according to the instructions. The quality of RNA was evaluated by an Agilent 2100 Bioanalyzer (Agilent Technologies, Palo Alto, CA, USA), and samples with an RNA integrity number (RIN) value above 9 were suitable for cDNA library construction. RNA-Seq libraries generated from the MCM10 CA1a and MCF10 DCIS cell lines (with duplicates for each sample type) were prepared with a VAHTS mRNA-Seq V2 Library Prep Kit for Illumina (Vazyme Biotech) and run on a HiSeq 2500 instrument (Illumina, San Diego, CA, USA). All the detailed steps are shown in the manufacturer’s commanded protocols. Alternative splicing analyses were performed by MISO software. DESeq2 (V1.6.3) in Bioconductor software was utilised for differential gene expression analyses. The DAVID database was used for gene ontology (GO) analyses. Gene set enrichment analysis (GSEA) was conducted by the GSEA software of the JAVA programme.

### Chromatin immunoprecipitation sequence (ChIP-seq) analysis

CHIP-seq assays were conducted with the SimpleChIP® Plus Enzymatic Chromatin IP Kit (Cell Signaling Technology, # 9005 S), and the DNA was purified with SimpleChIP® DNA Purification Buffers and Spin Columns (Cell Signaling Technology, #14209 S). All the detailed steps were performed according to the instructions of kit instructions. The DNA pulled down by a specific antibody was sent to Novogene Bioinformatics Institute (Beijing, China) for sequencing. Bowtie was utilised for mapping the FASTQ data to the human genome (hg19), and significant enrichments were identified with MACS2.0 using the broad peak mode with *P* ≤ 1 × 10^−5^ and FDR ≤0.05 as the cut-offs to cull peaks from the aligned results.

### Semiquantitative RT‒PCR, qPCR and CHIP-qPCR

Total RNA was extracted by TRIzol reagent (Invitrogen). HiScript II Q Select RT SuperMix for qPCR (Vazyme, R233-01) was used to reverse transcribe RNA into cDNA. The primers used for validating AS events were designed using SnapGene software (Version 4.3.6) according to the mRNA sequences on NCBI (Table. S1). The cDNA was amplified by Q5® High-Fidelity DNA Polymerase (NEB, M0491S) and separated by 2% agarose gel electrophoresis. For ChIP‒qPCR, ChIP was performed with the SimpleChIP® Plus Enzymatic Chromatin IP Kit (Cell Signaling Technology, # 9005 S), and the primers were designed according to published articles. AceQ qPCR SYBR Green Master Mix (Vazyme, Q111-03) was used for qPCR, and the GAPDH gene was used for normalisation. The detailed protocols are shown in the manufacturer’s recommended protocols.

### CCK-8, colony formation and transwell migration assays

For CCK-8 assays, the counted cells were diluted to 1×10^4^/ml, seeded in 96-well plates (100 µl per well) and three replicate wells in each group, and then inoculated with complete growth medium for 7 days. Cell Counting Kit (CCK-8) solution (Yeasen, #40203ES60) was added and incubated for 2 h every day, and a microplate reader was used to measure the absorbance at 450 nm. For colony formation, 1 × 10^3^ cells were seeded in six-well plates per well, and three replicate wells were inoculated with a complete growth medium for each group. After 14 days, the cells were fixed with methanol, stained with 0.05% crystal violet and photographed.

For transwell migration assays, Falcon® Transparent Inserts were purchased from Corning (#353097). MCF10 CA1a/DCIS cells (cultured 10 w) or MDA-MB-231 cells (cultured 5 w) were suspended in 200 µl serum-free media and added to the chamber. Then, 600 µl cell culture media with 20% serum was added to the lower layer of the chamber. MCF10 CA1a/DCIS cells were incubated for 24 h, and MDA-MB-231 cells were incubated for 7 h. After wiping away the cells that failed to migrate, the migrated cells were fixed with methanol, stained with 0.05% crystal violet, and imaged.

### Animal models

The experimental animals used in this article were 6- to 8-week-old female NOD. CB17-Prkdcscid/Shjh, which was purchased from Shanghai Jihui Laboratory Animal Care Co., Ltd. All animal experiments were given permissions from the Fudan Animal Ethics committee (approval number, 2017-031) and on the basis of the NIH Guide for the Care and Use of Laboratory Animals (http://oacu.od.nih.gov/regs/index.htm). MCF10 CA1a HNRNPU knockout (KO) and normal control (NC) cells (5 × 10^6^) suspended in 100 µl PBS were injected into the mammary fat pad of NOD/SCID mice. The KO1 group, KO2 group and NC group were randomly distributed into eight mice. The subcutaneous tumours were accessed by digital callipers every 5 days. After 28 days, the tumours were dissected, weighed, imaged and fixed for further IHC analyses. For metastasis models, 1 × 10^6^ MCF10 CA1a HNRNPU knockout (KO) and normal control (NC) cells suspended in 100 µl PBS were injected into the tail vein of NOD/SCID mice. After 28 days, the lungs were excised, and lung metastasis sites were counted and fixed for further IHC analyses.

### Immunohistochemical (IHC) staining

Tissue microarrays (TMAs) of 200 breast cancer patients were provided by the Department of Pathology of the Fudan University Shanghai Cancer Center (FUSCC). Paraffin sections were fabricated from the subcutaneous tumour tissues in the animal experiments, which were fixed with formalin, embedded in paraffin and then cut into 3-μm-thick sections. TMA sections and paraffin sections were baked in an oven at 70 °C for 1 h and dewaxed in xylene. Next, 100, 90 and 70% alcohol were used to hydrate the samples, and citric acid buffer (pH = 6.0) was utilised to retrieve antigen at 95 °C for 20 min. Finally, processed sections were incubated with antibodies according to the manufacturer’s instructions. The antibodies are listed in Table. S2. The TMA sections and paraffin sections were observed using an Olympus BX43 microscope. The HNRNPU score was comprehensively determined by the percentage of the stained area (1, 0–10%; 2, 10–50%; 3, 50–100%) and the intensity of positive staining (1, weak; 2, moderate; 3, strong).

### Clinical samples

Breast cancer specimens and adjacent normal tissues were acquired from breast cancer patients who underwent surgery at FUSCC. All experiments involving humans were conducted on the basis of the Declaration of Helsinki. Before the patients were enroled in the experiment, informed consent was provided, and the study was permitted by the Independent Ethical Committee/Institutional Review Board of Fudan University Shanghai Cancer Center.

### Statistical analysis

R software version 3.5.3, GraphPad Prism version 8.0 (GraphPad Software, Inc.) and SPSS version 25.0 (SPSS, Chicago, IL) were utilised for statistical analysis in this article. The data are expressed as the mean ± standard deviation from at least three independent experiments. An unpaired two-tailed Student’s *t*-test was performed to analyse continuous variables. The Kaplan‒Meier method and log-rank test were conducted to compare survival times. Pearson’s test was used to perform correlation analysis. All *p* values were two-sided, and a *P* value of less than 0.05 was considered statistically significant.

## Supplementary information

Figure Legends

Han BY et al. reproducibility checklist

Supplemental figures

Supplemental Table 1

Supplemental Table 2

Original Data File

## Data Availability

All data were available from the corresponding author upon reasonable request.
