# Supplementary material for: HNRNPU promotes the progression of triple-negative breast cancer via RNA transcription and alternative splicing mechanisms
Source: Cell Death Dis. 2022 Nov 8;13(11):940. doi: 10.1038/s41419-022-05376-6 (PMC9643420; doi:10.1038/s41419-022-05376-6)
Supplement: Supplementary file 1 — Figure Legends [file 41419_2022_5376_MOESM1_ESM.docx]

**Figure legends**

**Figure 1 CRISPR/CAS9 screening library analysis revealed that HNRNPU is related to breast cancer progression.**

1A Experimental outline of the CRISPR/CAS9 screening library.

2B Venn diagram of the top 200 genes in MCF10 CA1a and MCF10 DCIS cell lines based on negative screening.

1C Scatterplot of the top 200 candidate genes in our screening model cells. The top 200 genes are marked with different coloured dots.

1D Heatmap of the mRNA levels of the 43 overlapping candidate genes in MCF10A, MCF10 AT, MCF10 DCIS and MCF10 CA1a cells.

1E HNRNPU mRNA expression in breast cancer tissue and normal tissue in the TCGA database.

1F HNRNPU mRNA expression in breast cancer tissue and normal tissue in the FUSCC TNBC database.

1G HNRNPU expression across the four molecular subtypes of breast cancer and normal tissue in the TCGA datasets. The number of patients is shown in brackets. Whiskers indicate the minimum and maximum values.

1H HNRNPU expression across the four molecular subtypes of breast cancer and normal tissue in the METABRIC datasets. The number of patients is shown in brackets. Whiskers indicate the minimum and maximum values.

1I Kaplan–Meier analysis of RFS using the Kaplan–Meier plotter database.

1J Kaplan–Meier analysis of RFS using the FUSCC-TNBC database.

1K, 1L Representative images of HNRNPU IHC staining in TNBC patients and statistical analysis of HNRNPU expression according to the IHC score.

1L Kaplan–Meier analysis of RFS in TNBC patients. A log-rank test was used to determine the statistical significance between the low HNRNPU expression group and the high HNRNPU expression group.

**Figure 2** **HNRNPU promotes TNBC cell proliferation and migration in vitro in vivo.**

2A Western blot analyses of HNRNPU protein expression in MCF10 cells.

2B qPCR analyses of the HNRNPU mRNA levels in MCF10 cells.

2C Western blot analyses of HNRNPU protein expression in 8 breast cancer cell lines.

2D Western blot analysis of HNRNPU protein expression in 6 pairs of matched breast cancer specimens and adjacent normal breast tissues.

2E, F, G Western blot analyses of CRISPR-mediated knockout of HNRNPU in MDA-MB-231, MCF10 CA1a and MCF10 DCIS cells.

2H, I, J Cell proliferation analyses using the CCK-8 assay in MDA-MB-231, MCF10 CA1a and MCF10 DCIS cells.

2K Cell proliferation analyses using a colony formation assay in MDA-MB-231, MCF10 CA1a and MCF10 DCIS cells. Representative images of surviving colonies (left) and corresponding quantitative results (right) are shown.

2L Transwell migration assay of MDA-MB-231, MCF10 CA1a and MCF10 DCIS cells. Representative images of cell migration (left) and quantitative results (right) are shown.

2M Photographs of harvested tumours (left) and tumour growth curves (right) are shown.

2N Representative photographs of metastatic lung nodules (left), H&E-stained sections of lung tissues (middle) and quantitative results of nodules (right) are shown. Data are presented as the mean ± SEM. ***p< 0.001, **p< 0.01, *p< 0.05, n.s., not significant.

**Figure 3 HNRNPU interacts with DDX5.**

3A Heatmap of proteins identified from Flag-HNRNPU IP/MS analysis.

3B Heatmap of splicing factors and transcription factors identified from Flag-HNRNPU IP/MS analysis, Venn diagram of splicing factors and transcription factors identified from Flag-HNRNPU IP/MS analysis.

3C, 3D Coimmunoprecipitation analysis shows the interaction between HNRNPU and DDX5.

3E, 3F Coimmunoprecipitation analysis shows the interaction between endogenous HNRNPU and DDX5 in HEK293T cells.

3G Schematic diagram of HNRNPU domains and constructions of two HNRNPU mutants. All mutants were FLAG tagged.

3H Coimmunoprecipitation analysis shows the interaction between endogenous Flag-tagged HNRNPU truncation mutants and DDX5 in HEK293T cells.

3I, 3J Effects of HNRNPU expression on DDX5 protein levels in MCF10 CA1a cells by immunoblotting analysis.

3K, 3L Effects of DDX5 expression on HNRNPU protein levels in MCF10 CA1a cells by immunoblotting analysis.

**Figure 4 HNRNPU modulates alternative splicing events by interacting with DDX5**

4A Quantification of AS events after HNRNPU was knocked out or DDX5 was knocked down.

4B Venn diagram of the AS events regulated by HNRNPU and DDX5.

4C, 4D PSI profiles of SE and RI events identified in control and HNRNPU knockout cells (left). The coloured dots represent significantly upregulated (red) or downregulated (blue) events in HNRNPU knockout cells compared with control cells (left). [Pie](https://cn.bing.com/dict/search?q=pie&FORM=BDVSP6&cc=cn) [chart](https://cn.bing.com/dict/search?q=chart&FORM=BDVSP6&cc=cn) shows the quantification of upregulated (pink) or downregulated (purple) SE and RI events after HNRNPU was knocked out (right).

4E, 4F PSI profiles of SE and RI events identified in control and DDX5 knockdown cells (left). The coloured dots represent significantly upregulated (red) or downregulated (blue) events in DDX5 knockdown cells compared with control cells (left). [Pie](https://cn.bing.com/dict/search?q=pie&FORM=BDVSP6&cc=cn) [chart](https://cn.bing.com/dict/search?q=chart&FORM=BDVSP6&cc=cn) shows the quantification of upregulated (pink) or downregulated (purple) SE and RI events after DDX5 was knocked down (right).

4G Representative RT‒PCR validation of HNRNPU-regulated SE events and DDX5-regulated SE events. The structure of each isoform is illustrated in the diagrams. Individual data points are presented (n=3; ***p< 0.001, **p< 0.01, *p< 0.05; Student’s t test).

4H Representative RT‒PCR validation of HNRNPU-regulated RI events and DDX5-regulated RI events. The structure of each isoform is illustrated in the diagrams. Individual data points are presented (n=3; ***p< 0.001, **p< 0.01, *p< 0.05; Student’s t test).

4I HNRNPU knockout increases the MCM10 splicing for intron retention and decreases MCM10 mRNA expression. Quantitative real-time PCR analysis of MCM10 mRNA expression and intron retention value.

4J DDX5 knockdown increases the MCM10 splicing for intron retention and decreases MCM10 mRNA expression. Quantitative real-time PCR analysis of MCM10 mRNA expression and intron retention value.

4K Schematics of the MCM10 alternative splicing pattern regulated by HNRNPU and DDX5. The first stop codon in the intron is indicated with red letters.

4L HNRNPU knockout and control MCF10 CA1a cells were transfected with control siRNA and UPF1 siRNA. MCM10 mRNA expression was examined by Quantitative real-time PCR.

**Figure 5 HNRNPU regulates transcription by interacting with DDX5.**

5A Volcano plot of differentially expressed genes after the knockout of HNRNPU (left). Red and blue indicate high and low expression, respectively. Heatmap representing the unsupervised hierarchical clustering of mRNA expression levels in HNRNPU knockout and control MCF10 CA1a cells (right).

5B Volcano plot of differentially expressed genes after the knockdown of DDX5 (left). Red and blue indicate high and low expression, respectively. Heatmap representing the unsupervised hierarchical clustering of mRNA expression levels in DDX5 knockdown and control MCF10 CA1a cells (right).

5C GSEA results were plotted to visualize the correlation between the expression of HNRNPU and related carcinogenic pathways in MCF10 CA1a cells.

5D [Pie](https://cn.bing.com/dict/search?q=pie&FORM=BDVSP6&cc=cn) [chart](https://cn.bing.com/dict/search?q=chart&FORM=BDVSP6&cc=cn) shows the genomic distribution of CHIP-Seq peaks for HNRNPU.

5E Average genome-wide occupancies of HNRNPU along the transcription unit. The gene body length was aligned 3000 bp upstream and downstream of the TSS.

5F [Pie](https://cn.bing.com/dict/search?q=pie&FORM=BDVSP6&cc=cn) [chart](https://cn.bing.com/dict/search?q=chart&FORM=BDVSP6&cc=cn) shows the genomic distribution of CHIP-Seq peaks for DDX5.

5G Average genome-wide occupancies of DDX5 along the transcription unit. The gene body length was aligned 3000 bp upstream and downstream of the TSS.

5H Venn diagram illustrating the overlap of HNRNPU- and DDX5-occupied genes.

5I Heatmap representing the mRNA expression levels of 25 overlapping genes (in Figure 5H) in HNRNPU knockout and DDX5 knockdown cells.

5J HNRNPU and DDX5 enrichment on the Lmo4 promoter was analysed via ChIP‒qPCR analysis.

5K, 5L HNRNPU knockout decreases LMO4 mRNA expression. Quantitative real-time PCR analysis of HNRNPU and LMO4 mRNA expression.

5M, 5N DDX5 knockdown decreases LMO4 mRNA expression. Quantitative real-time PCR analysis of DDX5 and LMO4 mRNA expression.

**6. HNRNPU activates the Wnt/β-Catenin pathway and PI3K/Akt/mTOR pathway by regulating MCM10 and LMO4**

6A Western blot analysis of endogenous HNRNPU knockout MCF10 CA1a cells stably expressing exogenous MCM10-FLAG.

6B Cell proliferation assays of the cells described in Figure 6A.

6C, 6D, 6E Colony formation assays and Transwell assays of the cells described in Figure 6A. Representative images (6C) and quantitative results (6D, 6E) are shown.

6F MCF10 CA1a cells were transfected with two different shRNAs targeting LMO4 (LMO4-sh1, LMO4-sh2) or negative control shRNA (Ctrl-sh). Cells were analysed by immunoblotting after 48 h of transfection.

6G Cell proliferation assays of the cells described in Figure 6F.

6H, 6I Colony formation assays and Transwell assays of the cells described in Figure 6F. Representative images (H) and quantitative results (6I) are shown. Data are presented as the mean ± SEM. ***p< 0.001, **p< 0.01, *p< 0.05, n.s., not significant.

6J Western blot analysis showing the levels of P-β-catenin, c-Myc, CyclinD1, E-cadherin, MMP-2, MMP-9, Snail and Slug when the expression of MCM10 was decreased.

6K Western blot analysis showing the levels of MCM10, P-β-catenin, c-Myc, Cyclin D1, E-cadherin, MMP-2, MMP-9, Snail and Slug when the expression of HNRNPU was decreased.

6 L Western blot analysis showing the levels of P-PI3K, PI3K, P-akt, Akt, P-mTOR and m-TOR when the expression of LMO4 was decreased.

6 M Western blot analysis showing the levels of LMO4, P-PI3K, PI3K, P-akt, Akt, P-mTOR and m-TOR when the expression of HNRNPU was decreased.

**Figure 7 The HNRNPU-DDX5 complex is related to poor breast cancer prognosis, and the underlying molecular mechanism model was clarified**

7A Representative images of immunohistochemical staining as indicated in the xenografted tumours.

7B The proposed working model of this study.
