# Supplementary material for: HNRNPU promotes the progression of triple-negative breast cancer via RNA transcription and alternative splicing mechanisms
Source: Cell Death Dis. 2022 Nov 8;13(11):940. doi: 10.1038/s41419-022-05376-6 (PMC9643420; doi:10.1038/s41419-022-05376-6)
Supplement: Supplementary file 3 — Supplemental figures [file 41419_2022_5376_MOESM3_ESM.pdf]

Fig.S1

A

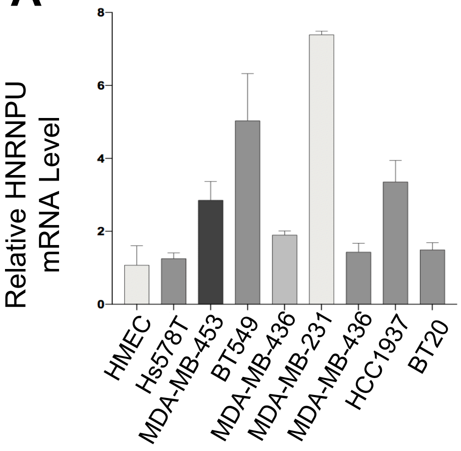

B

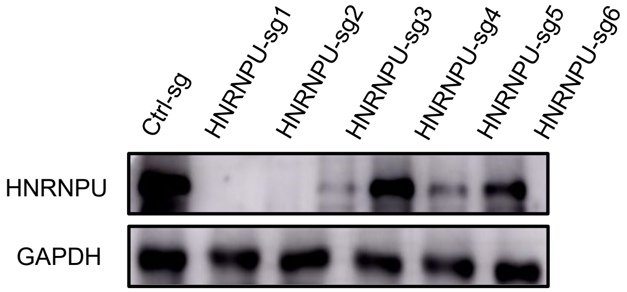

Fig.S1

A. qPCR analyses of the HNRNPU mRNA levels in HMEC and 8 breast cancer cell lines.

B. Western blot analyses of CRISPR-mediated Knock-out of HNRNPU.

Fig.S2

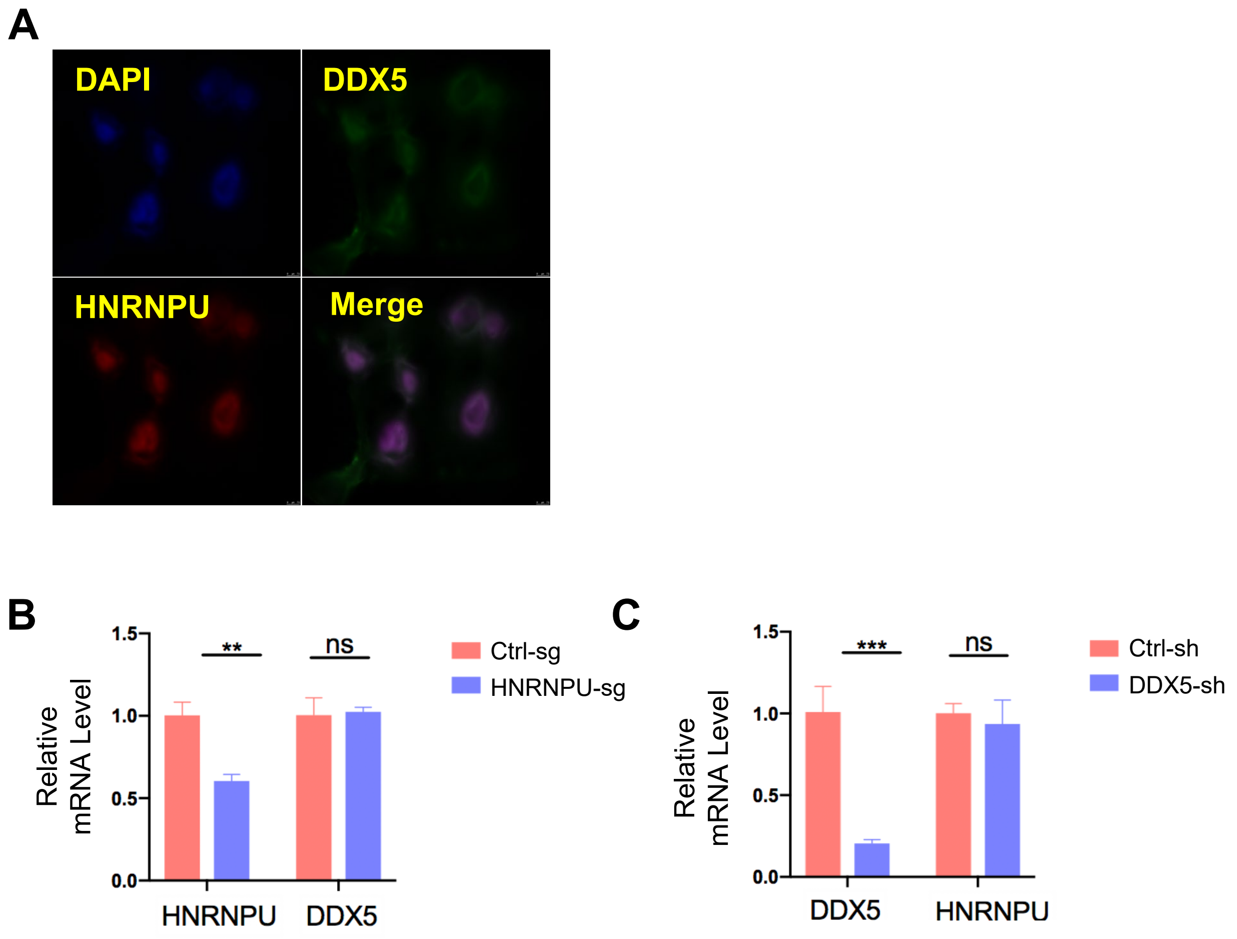

Fig.S2

A. Immunofluorescence staining of DAPI, HNRNPU and DDX5.

B. Quantitative real-time PCR analysis of DDX5 mRNA when HNRNPU was knocked out.

C. Quantitative real-time PCR analysis of HNRNPU mRNA when DDX5 was knocked down.

Fig.S3

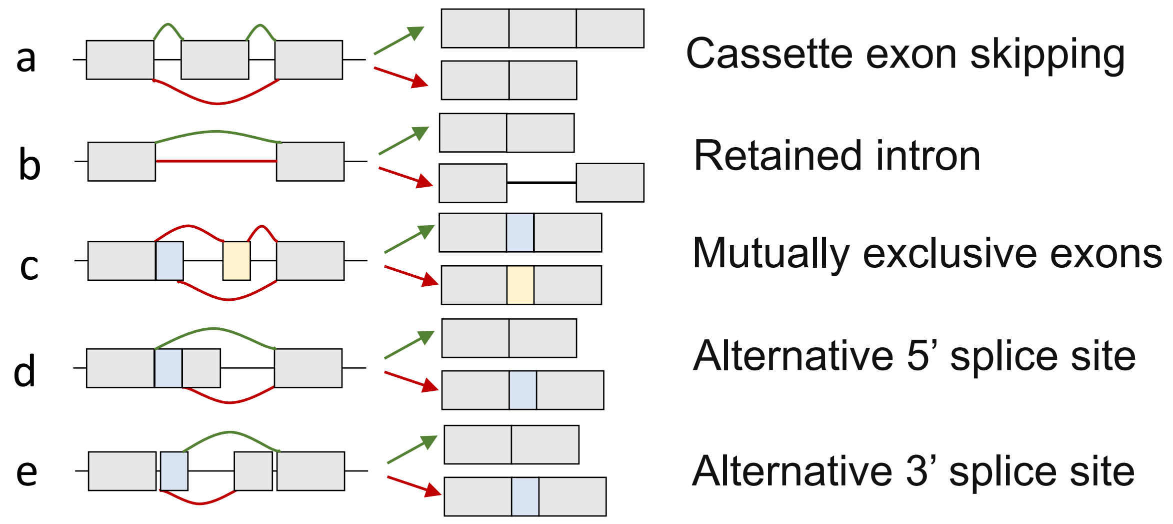

Fig.S3 Schematic depicting the five common modes of alternative splicing events: skipped exon (SE), retained intron (RI), mutually exclusive exon (MXE), alternative 5’ splice site (A5SS), and alternative 3’ splice site (A3SS). Shown on the right are the mature mRNA transcripts derived from each event. The green line represents normal splicing event, and the red line represents abnormal splicing event.

Fig.S4

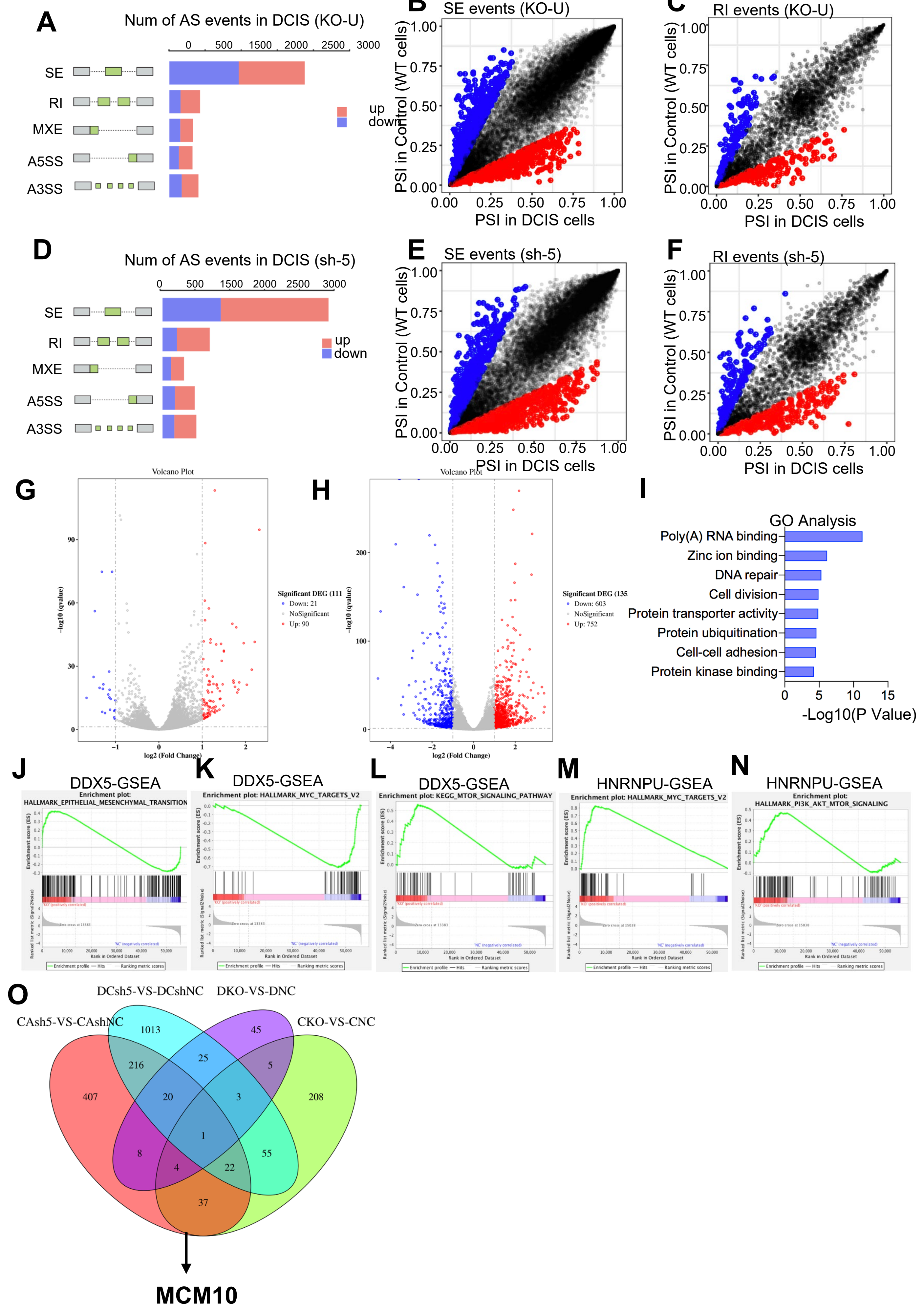
A
Num of AS events in DCIS (KO-U)
SE
RI
MXE
A5SS
A3SS
up
down
B
SE events (KO-U)
PSI in Control (WT cells)
PSI in DCIS cells
C
RI events (KO-U)
D
Num of AS events in DCIS (sh-5)
E
SE events (sh-5)
F
RI events (sh-5)
G
Volcano Plot
Significant DEG (111
Down: 21
NoSignificant
Up: 90
-log10 (qvalue)
log2 (Fold Change)
H
Significant DEG (135
Down: 603
Up: 752
I
GO Analysis
Poly(A) RNA binding
Zinc ion binding
DNA repair
Cell division
Protein transporter activity
Protein ubiquitination
Cell-cell adhesion
Protein kinase binding
-Log10(P Value)
J
DDX5-GSEA
HALLMARK_EPITHELIAL_MESENCHYMAL_TRANSITION
K
DDX5-GSEA
Enrichment plot: HALLMARK_MYC_TARGETS_V2
L
DDX5-GSEA
Enrichment plot: KEGG_MTOR_SIGNALING_PATHWAY
M
HNRNPU-GSEA
Enrichment plot: HALLMARK_MYC_TARGETS_V2
N
HNRNPU-GSEA
HALLMARK_PI3K_AKT_MTOR_SIGNALING
O
DCsh5-VS-DCshNC
DKO-VS-DNC
CAsh5-VS-CAshNC
CKO-VS-CNC
1013
45
216
25
5
407
20
3
208
1
8
55
4
22
37
MCM10

Fig.S4

A. Quantification of the upregulated (pink) or downregulated (purple) AS events in HNRNPU knock-out MCF10 DCIS cells.

B. PSI profiles of SE events identified in control and HNRNPU knock-out MCF10 DCIS cells.

C. PSI profiles of RI events identified in control and HNRNPU knock-out MCF10 DCIS cells.

D. Quantification of the upregulated (pink) or downregulated (purple) AS events in DDX5 knock-down MCF10 DCIS cells.

E. PSI profiles of SE events identified in control and DDX5 knock-down MCF10 DCIS cells.

F. PSI profiles of RI events identified in control and DDX5 knock-down MCF10 DCIS cells. The colored dots represent significantly upregulated (red) or downregulated (blue) event (B, C, E, F).

G, H Volcano plot of differentially expressed genes after the knock-out of HNRNPU (G) and the knock-down of DDX5 (H) in MCF10 DCIS cells. Red and blue indicate high and low expression, respectively.

I. Gene ontology analysis of HNRNPU-regulated AS targets. Fisher exact P values were plotted for each enriched functional category.

J, K, L, M, N GSEA results were plotted to visualize the correlation between the expression of HNRNPU and related carcinogenic pathways.

O. Venn diagram illustrates the overlap of HNRNPU and DDX5 regulated genes in MCF10 DCIS and MCF10 CA1a cells .

Fig.S5

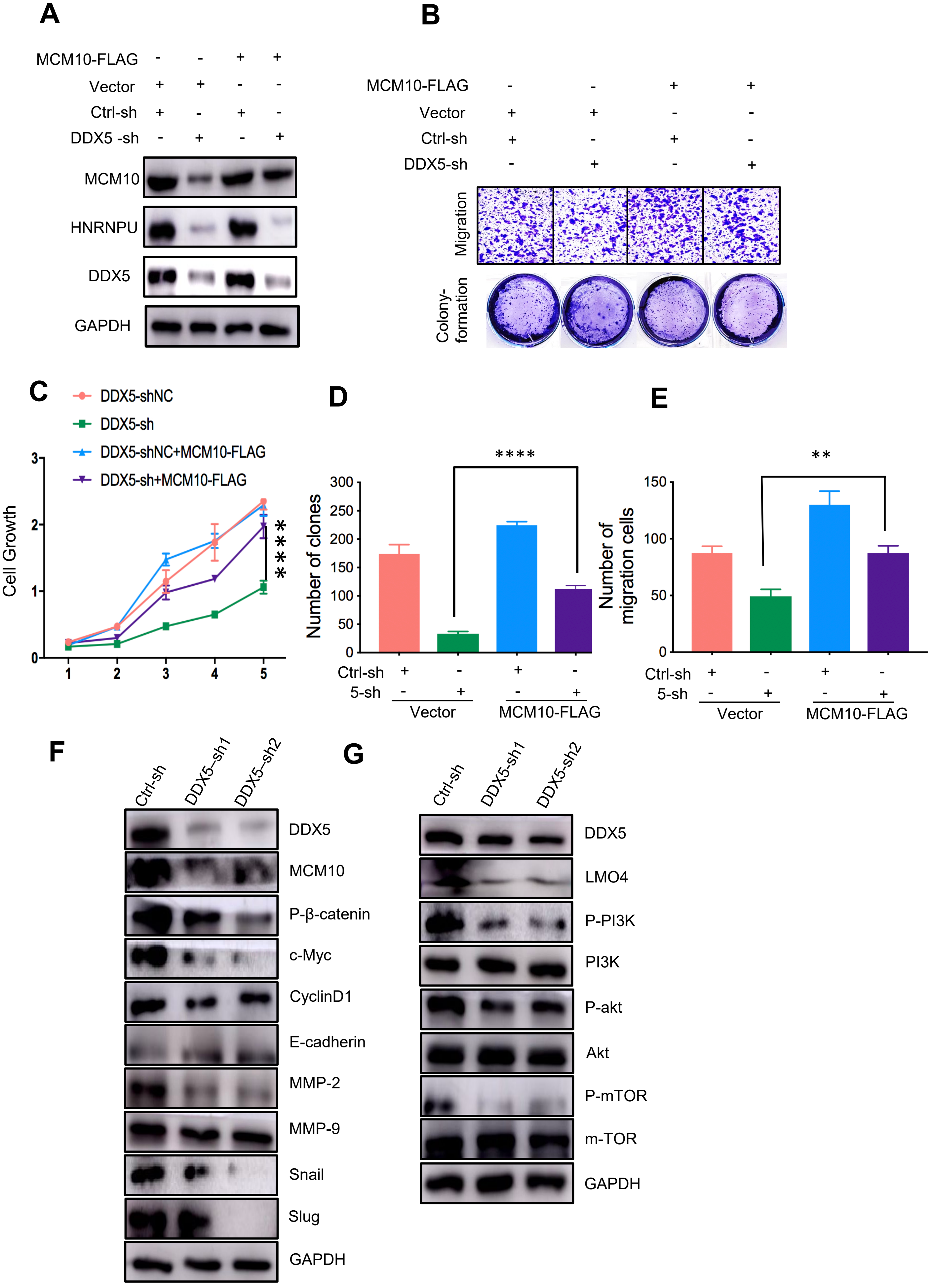
A
MCM10-FLAG - - + +
Vector + + - -
Ctrl-sh + - + -
DDX5 -sh - + - +
MCM10
HNRNPU
DDX5
GAPDH
B
MCM10-FLAG - - + +
Vector + + - -
Ctrl-sh + - + -
DDX5-sh - + - +
Migration
Colony-formation
C
DDX5-shNC
DDX5-sh
DDX5-shNC+MCM10-FLAG
DDX5-sh+MCM10-FLAG
Cell Growth
0
1
2
3
1 2 3 4 5
****
D
Number of clones
0
50
100
150
200
250
300
****
Ctrl-sh + - + -
5-sh - + - +
Vector
MCM10-FLAG
E
Number of migration cells
0
50
100
150
**
Ctrl-sh + - + -
5-sh - + - +
Vector
MCM10-FLAG
F
Ctrl-sh
DDX5-sh1
DDX5-sh2
DDX5
MCM10
P-β-catenin
c-Myc
CyclinD1
E-cadherin
MMP-2
MMP-9
Snail
Slug
GAPDH
G
Ctrl-sh
DDX5-sh1
DDX5-sh2
DDX5
LMO4
P-PI3K
PI3K
P-akt
Akt
P-mTOR
m-TOR
GAPDH

Fig.S5

A. Western blot analysis of endogenous DDX5 knock down MCF10 CA1a cells with stable expression of exogenous MCM10-FLAG.

B, D, E Colony formation assays and Trans-well assays of the cells described in Fig. S5A. Representative images (S5B) and quantitative results (S6D, S6E) are shown.

C. Cell proliferation assays of the cells described in Fig. S5A.

F. Western blot analysis showing the levels of MCM10, P-β-catenin, c-Myc, CyclinD1, E-cadherin, MMP-2, MMP-9, Snail and Slug when the expression of HNRNPU was depressed.

G. Western blot analysis showing the levels of LMO4, P-PI3K, PI3K, P-akt, Akt, P-mTOR and m-TOR when the expression of DDX5 was depressed.

Fig.S6

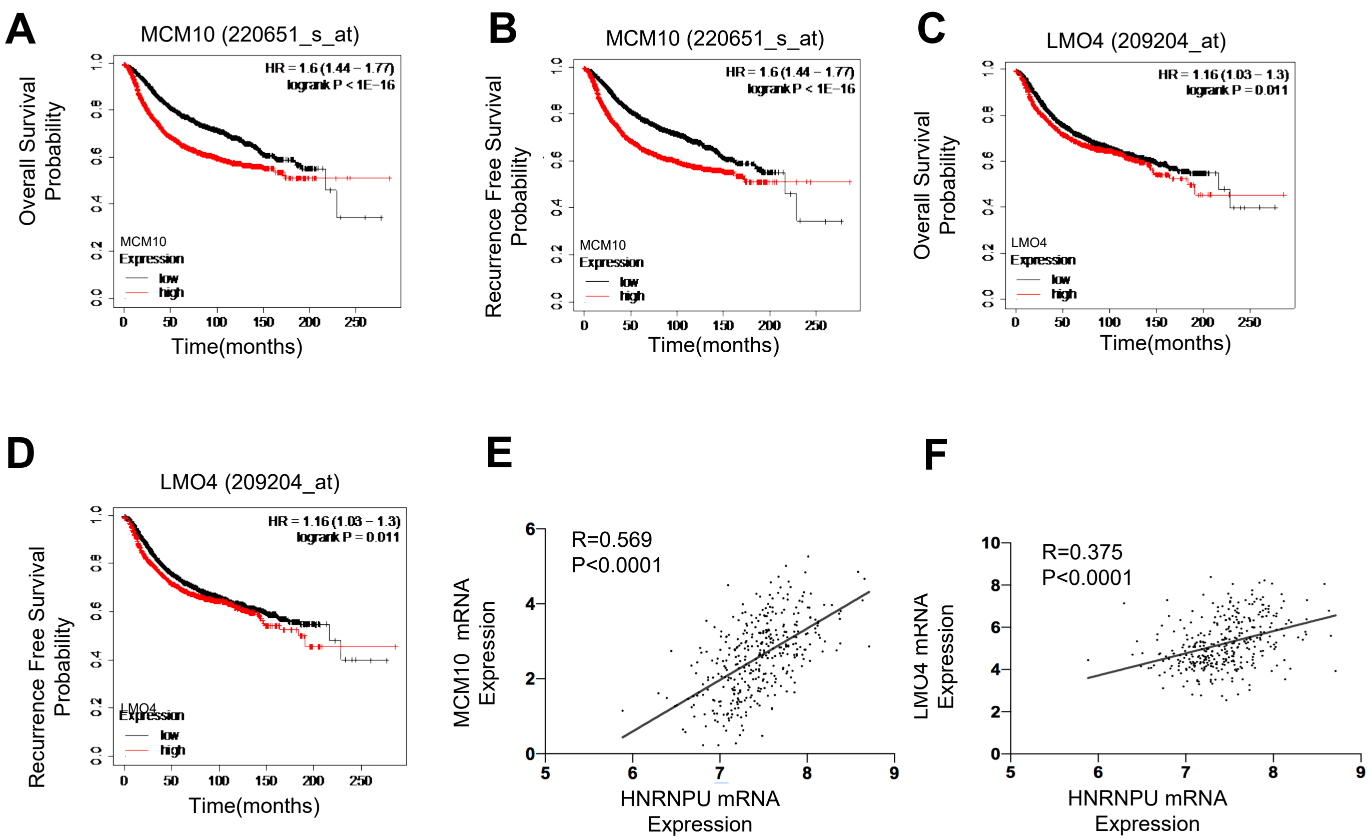

Fig.S6 A Kaplan–Meier analysis of OS based on MCM10 expression using the Kaplan–Meier plotter database.

B Kaplan–Meier analysis of RFS based on MCM10 expression using the Kaplan–Meier plotter database.

C Kaplan–Meier analysis of OS based on LMO4 expression using the Kaplan–Meier plotter database.

D Kaplan–Meier analysis of RFS based on LMO4 expression using the Kaplan–Meier plotter database.

E The scatterplot shows the correlation between MCM10 and HNRNPU mRNA expression levels.

F The scatterplot shows the correlation between LMO4 and HNRNPU mRNA expression levels.
