# Supplementary material for: HNRNPU promotes the progression of triple-negative breast cancer via RNA transcription and alternative splicing mechanisms
Source: Cell Death Dis. 2022 Nov 8;13(11):940. doi: 10.1038/s41419-022-05376-6 (PMC9643420; doi:10.1038/s41419-022-05376-6)

Full and uncropped western blot for Figure 2A

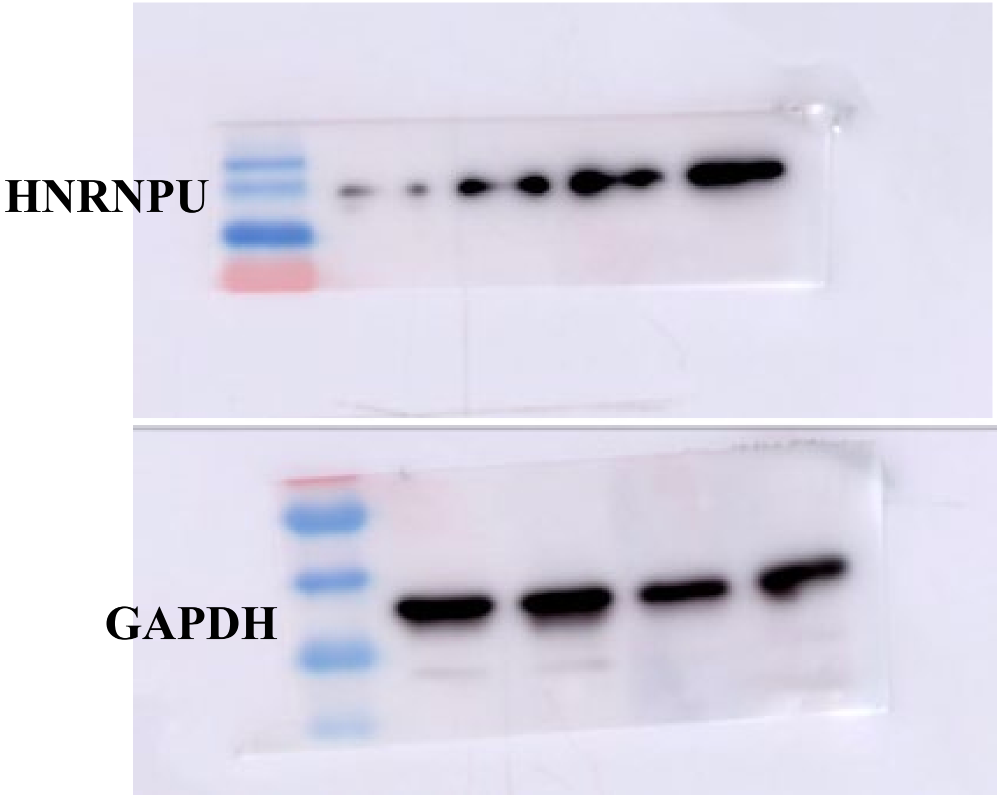

Full and uncropped western blot for Figure 2B

HNRNPU

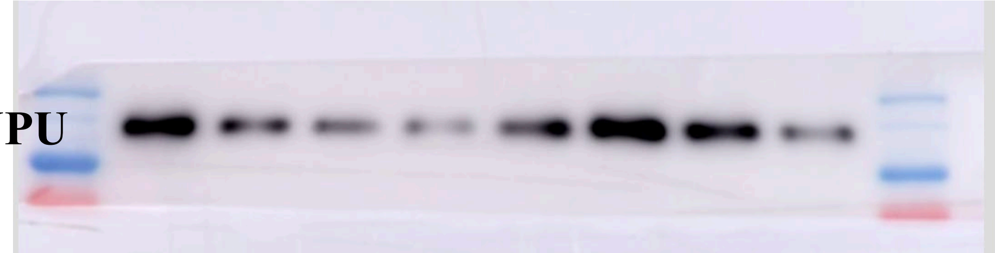

GAPDH

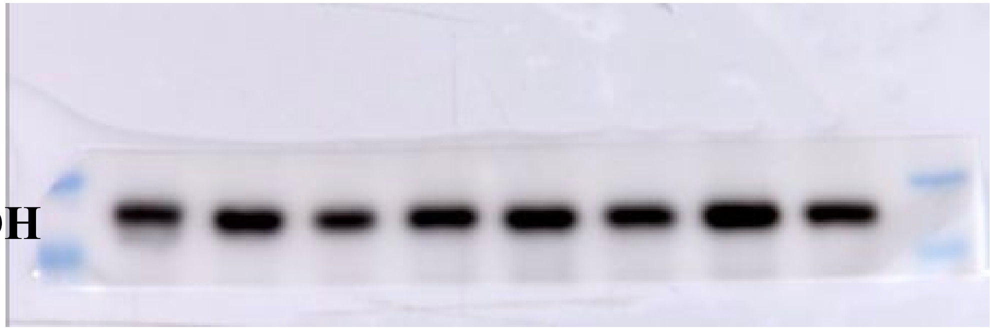

Full and uncropped western blot for Figure 2D

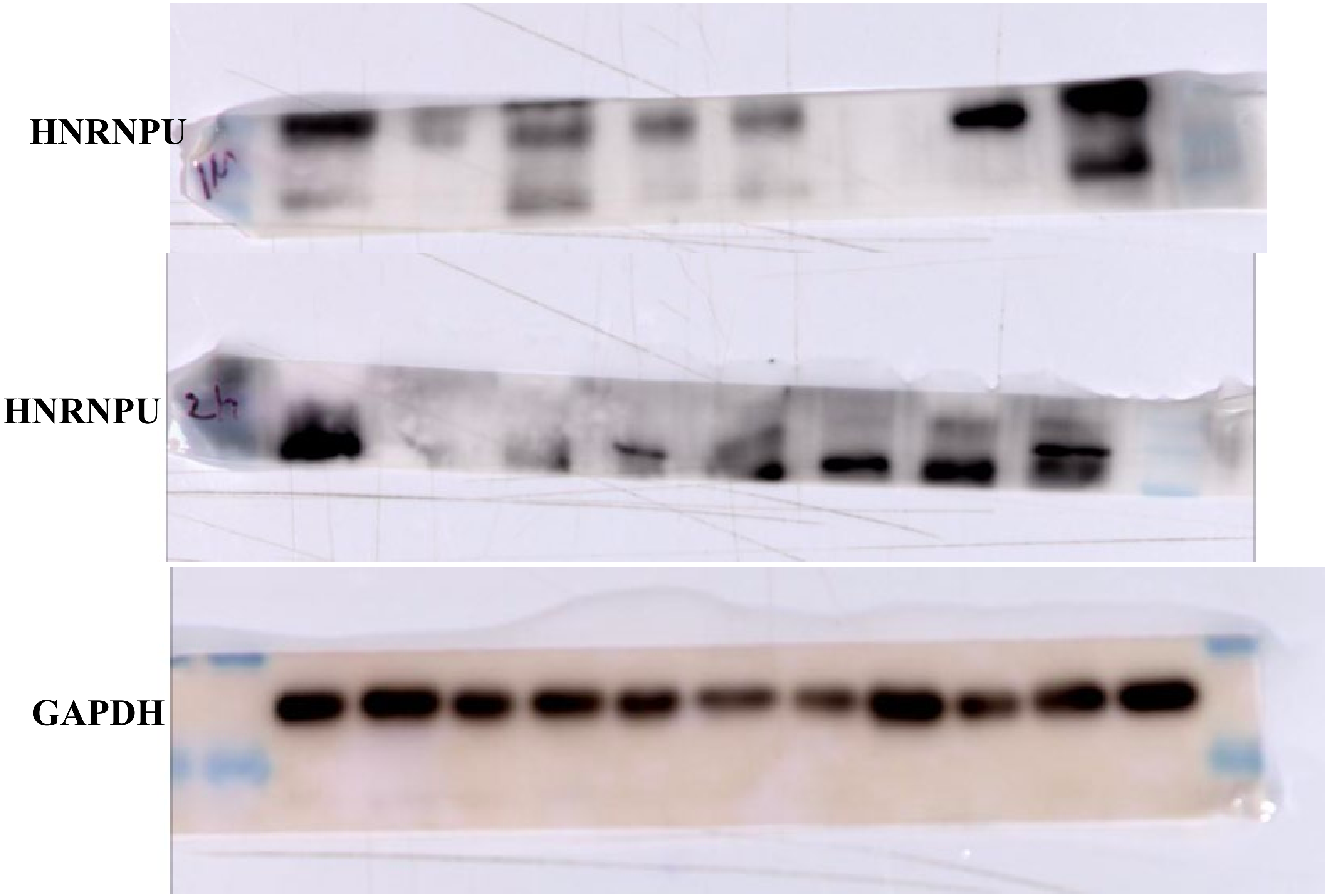

Full and uncropped western blot for Figure 2E, 2F, 2G

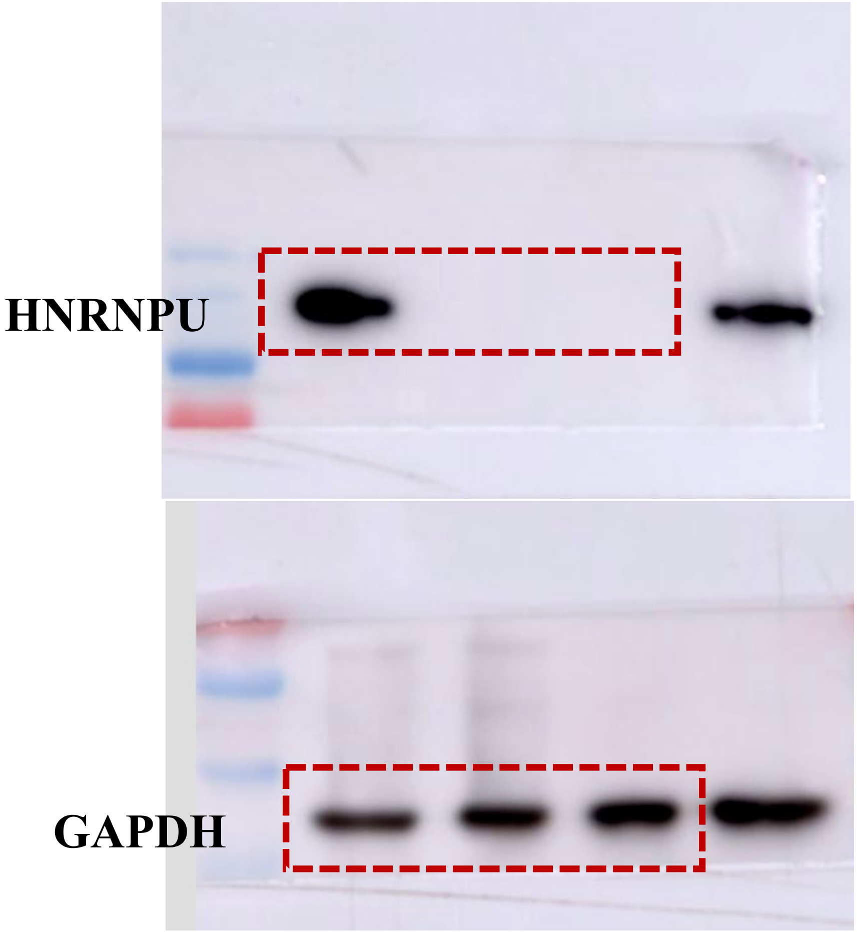

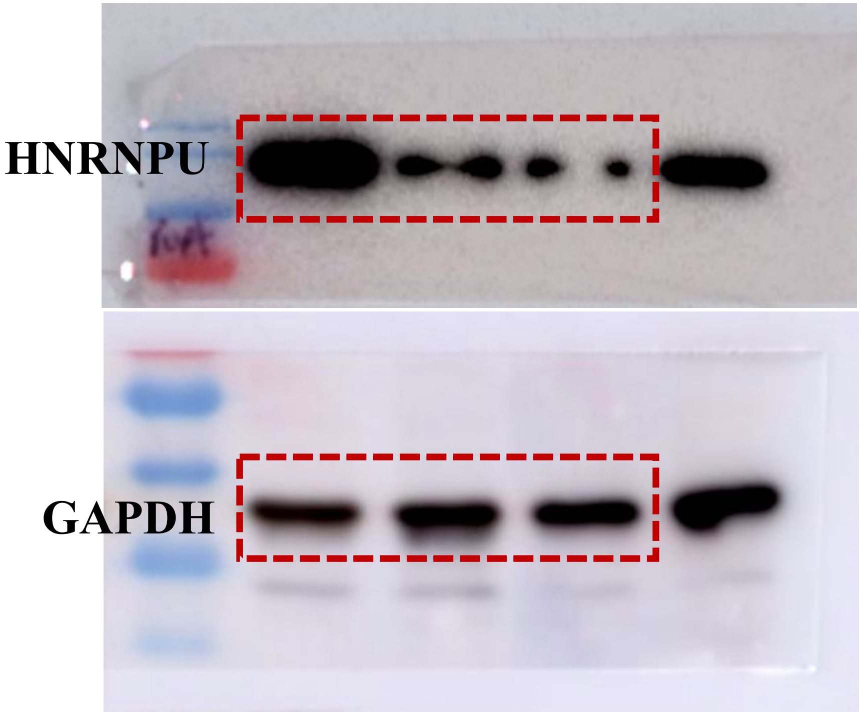

Full and uncropped western blot for Figure 3C,3D

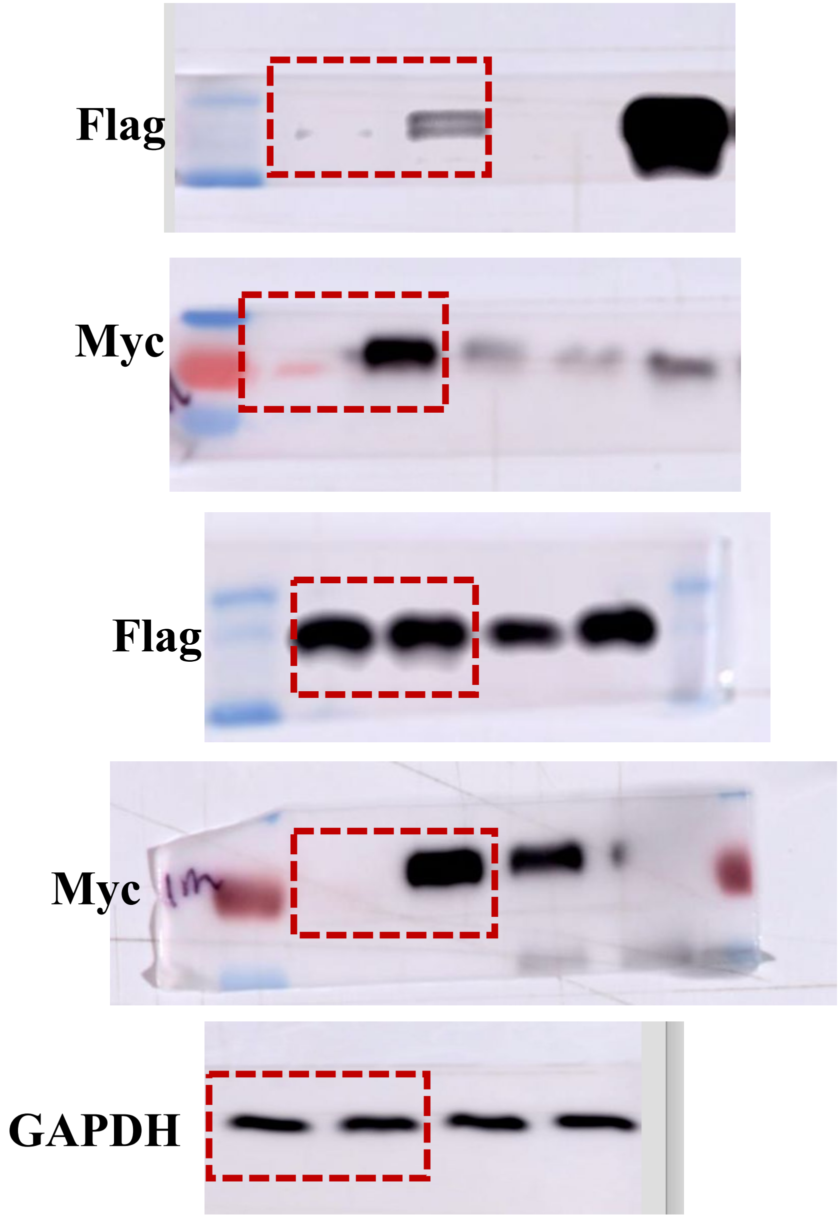

3C

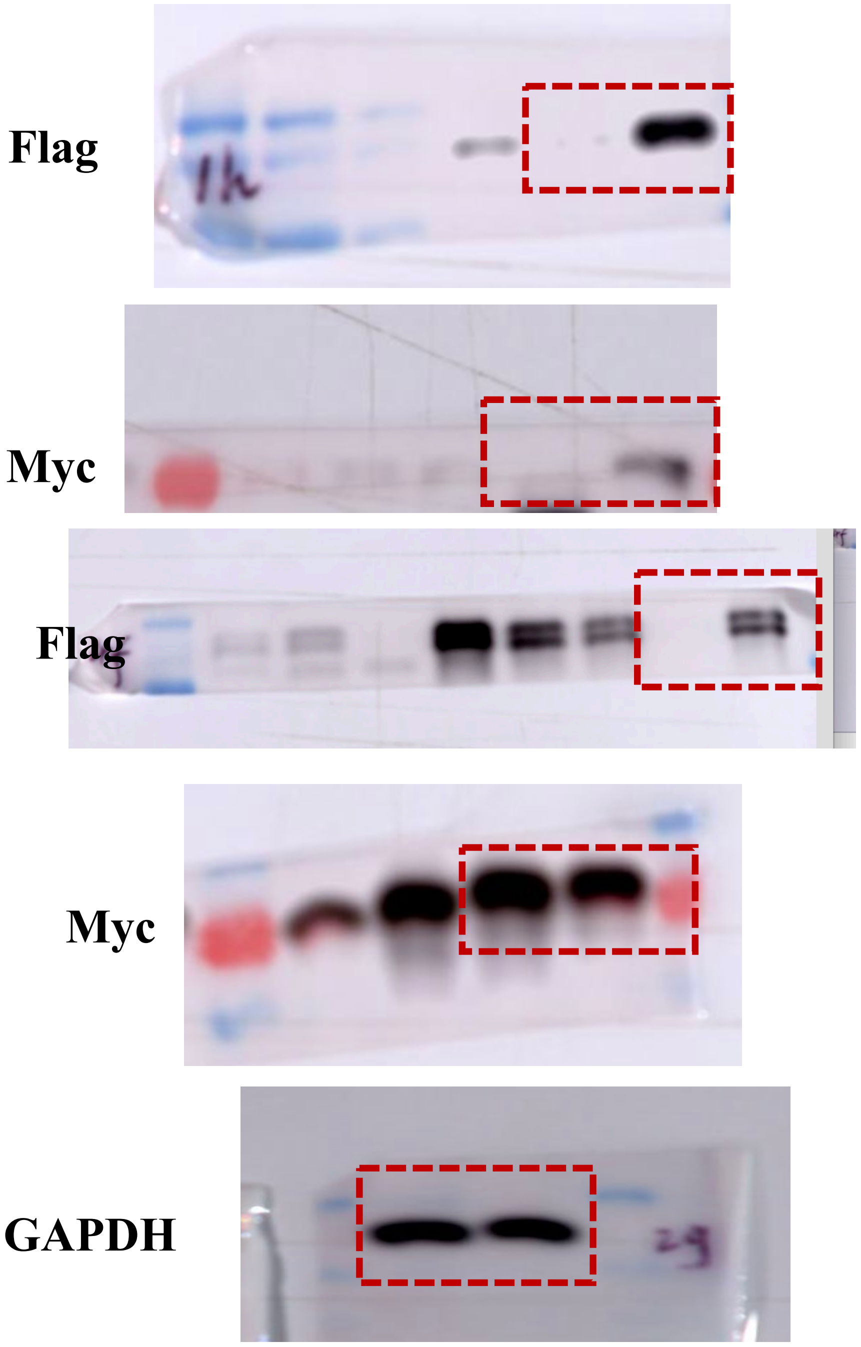

3D

Full and uncropped western blot for Figure 3E,3F

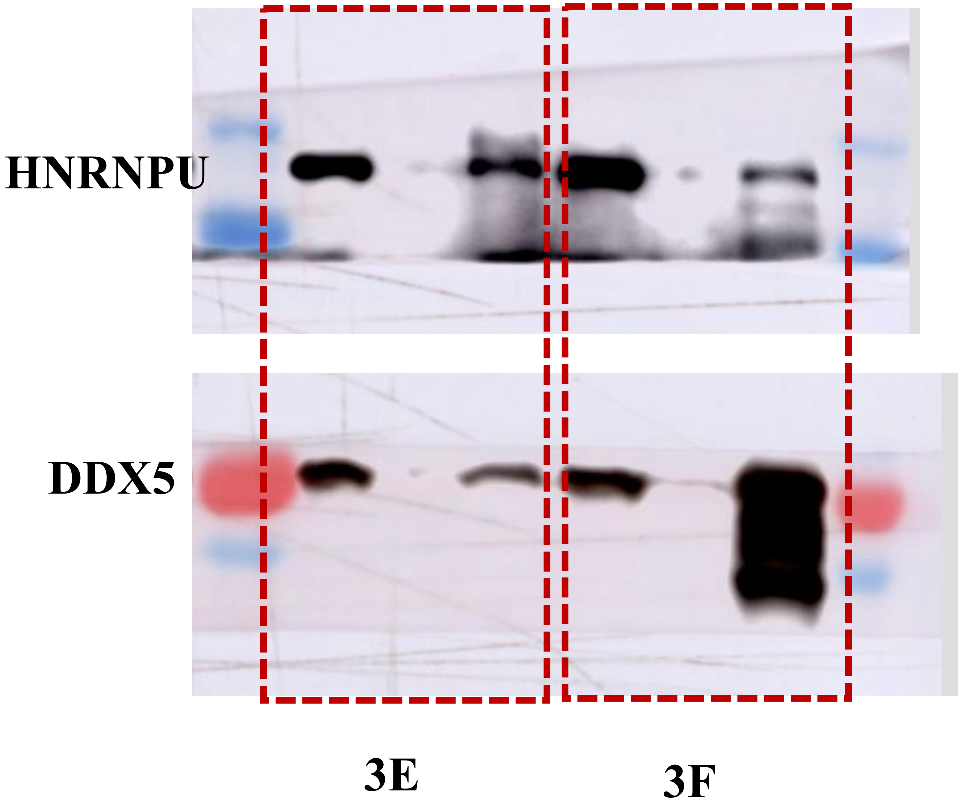

Full and uncropped western blot for Figure 3H

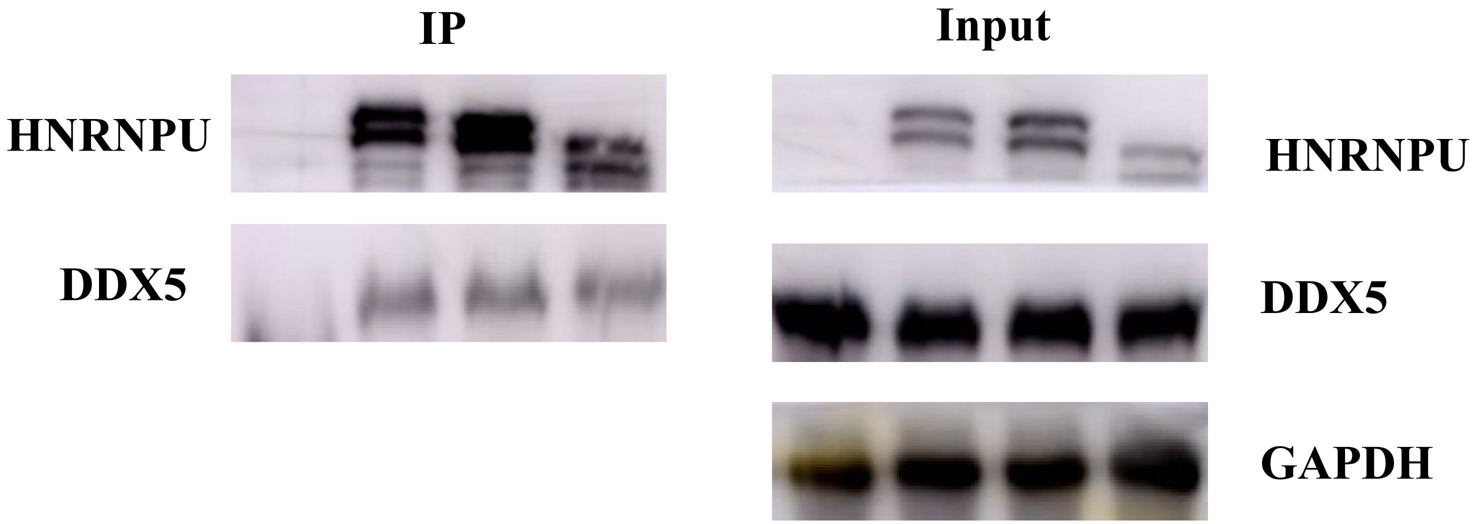

Full and uncropped western blot for Figure 3I, 3J

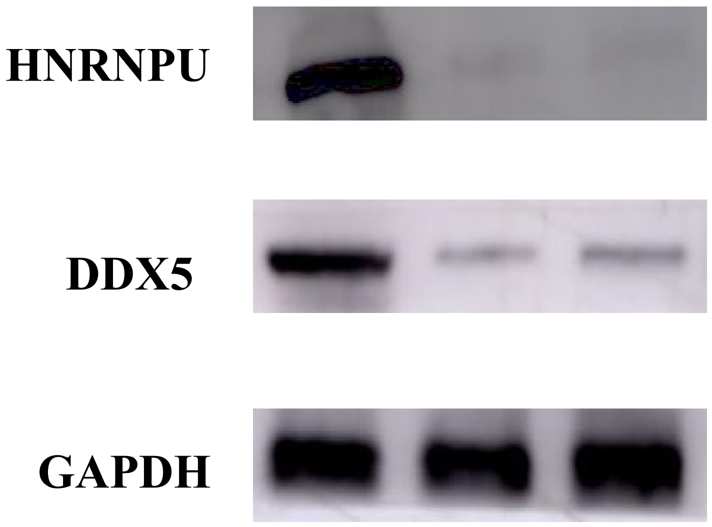

3I

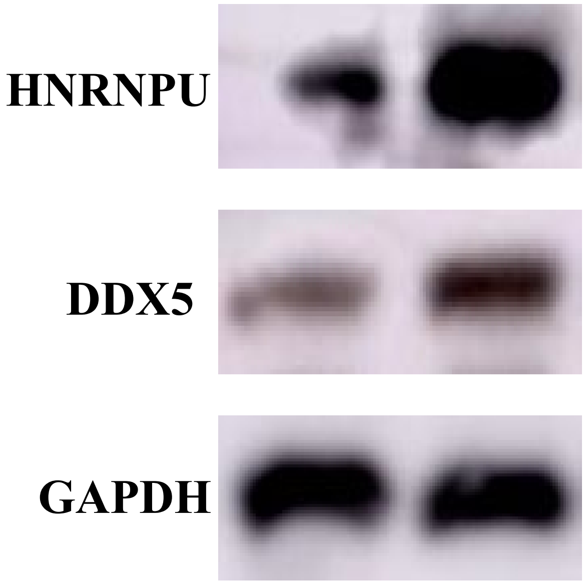

3J

Full and uncropped western blot for Figure 3K, 3L

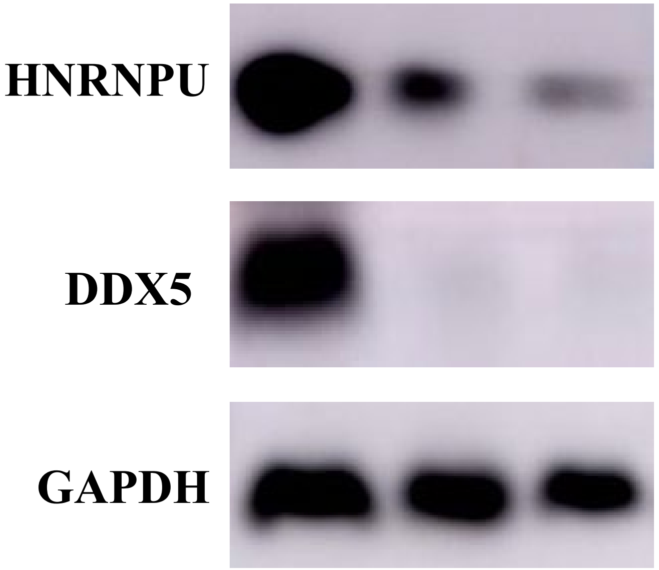

3K

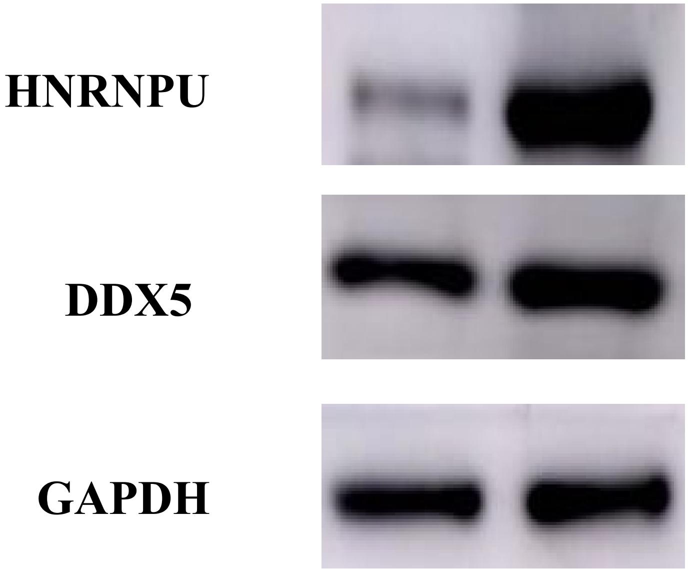

3L

Full and uncropped western blot for Figure 6A, 6F

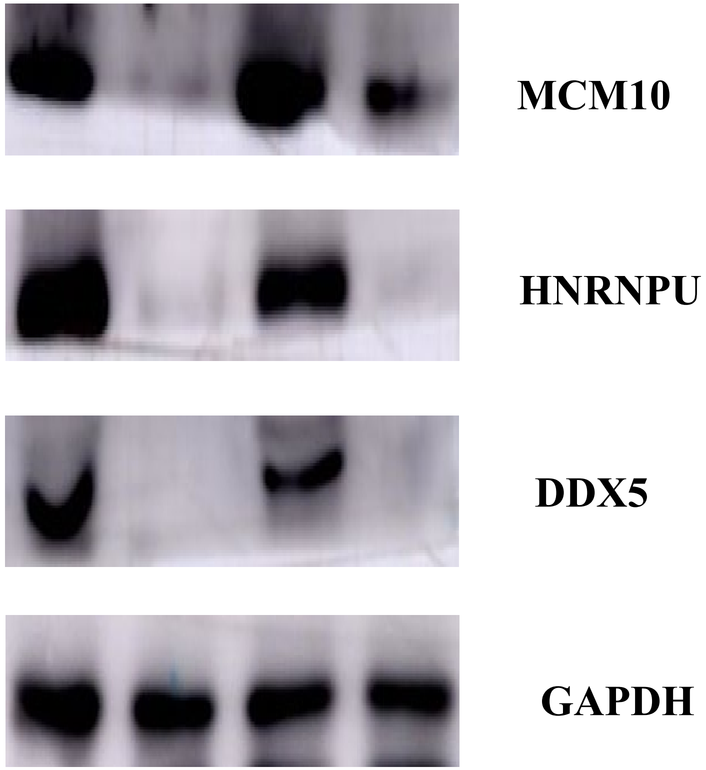

6A

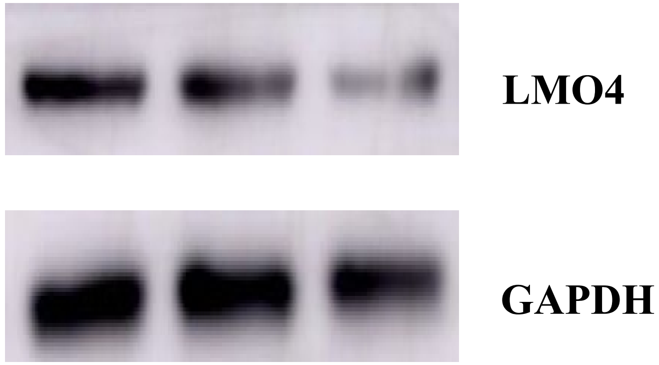

6F

Full and uncropped western blot for Figure 6J

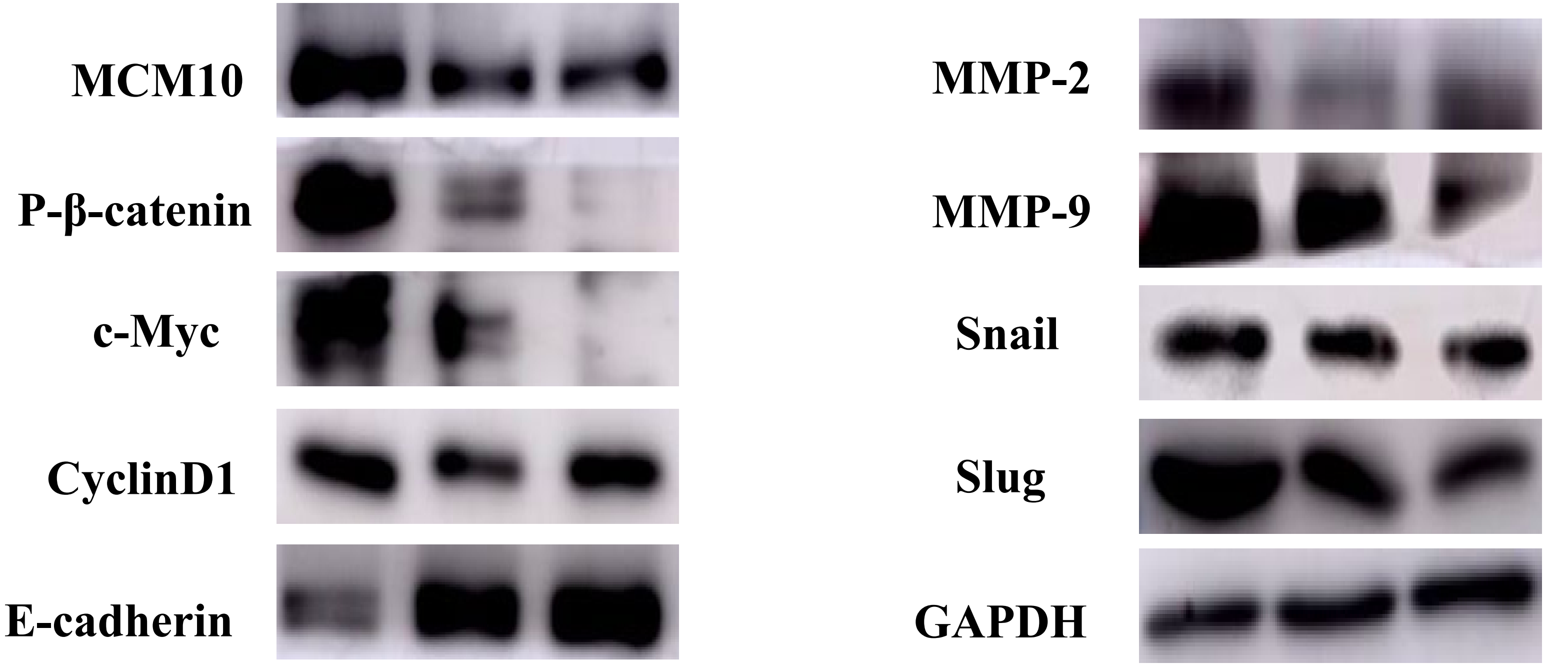

Full and uncropped western blot for Figure 6K

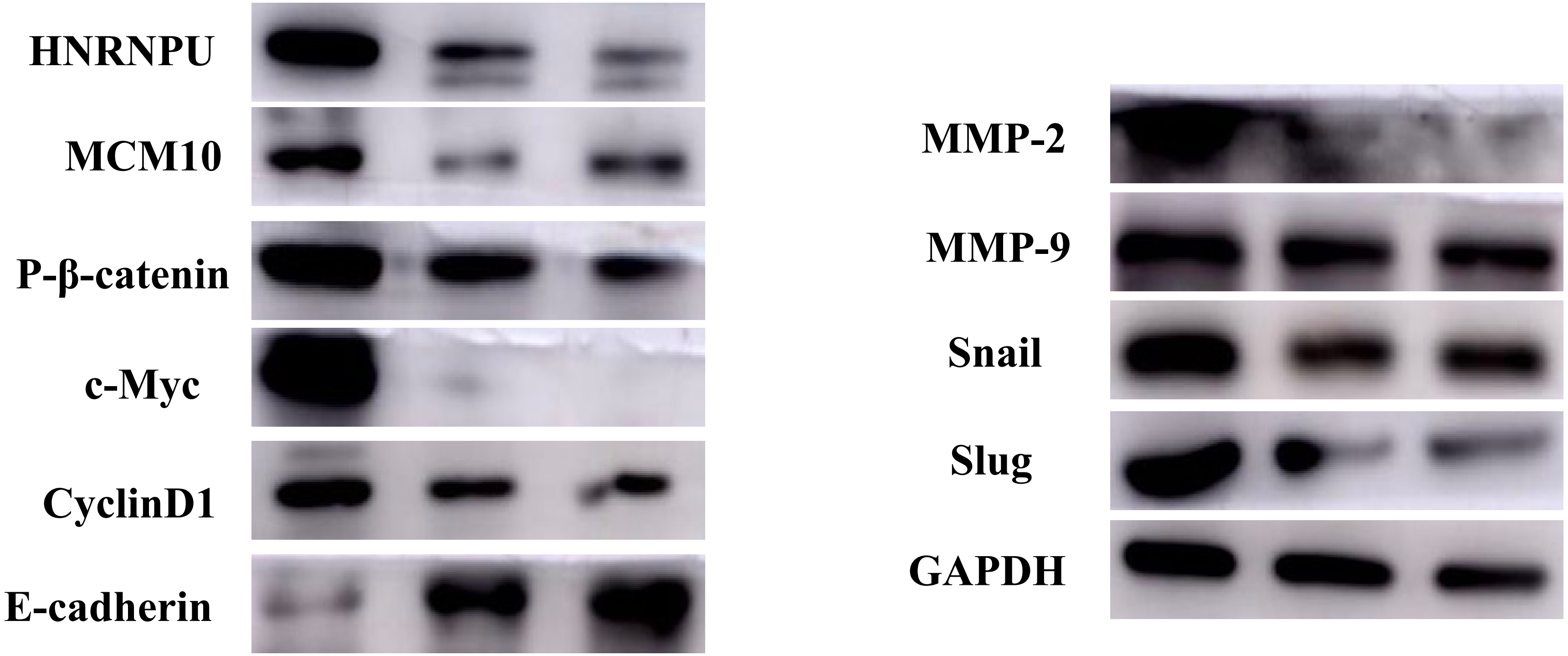

Full and uncropped western blot for Figure 6L

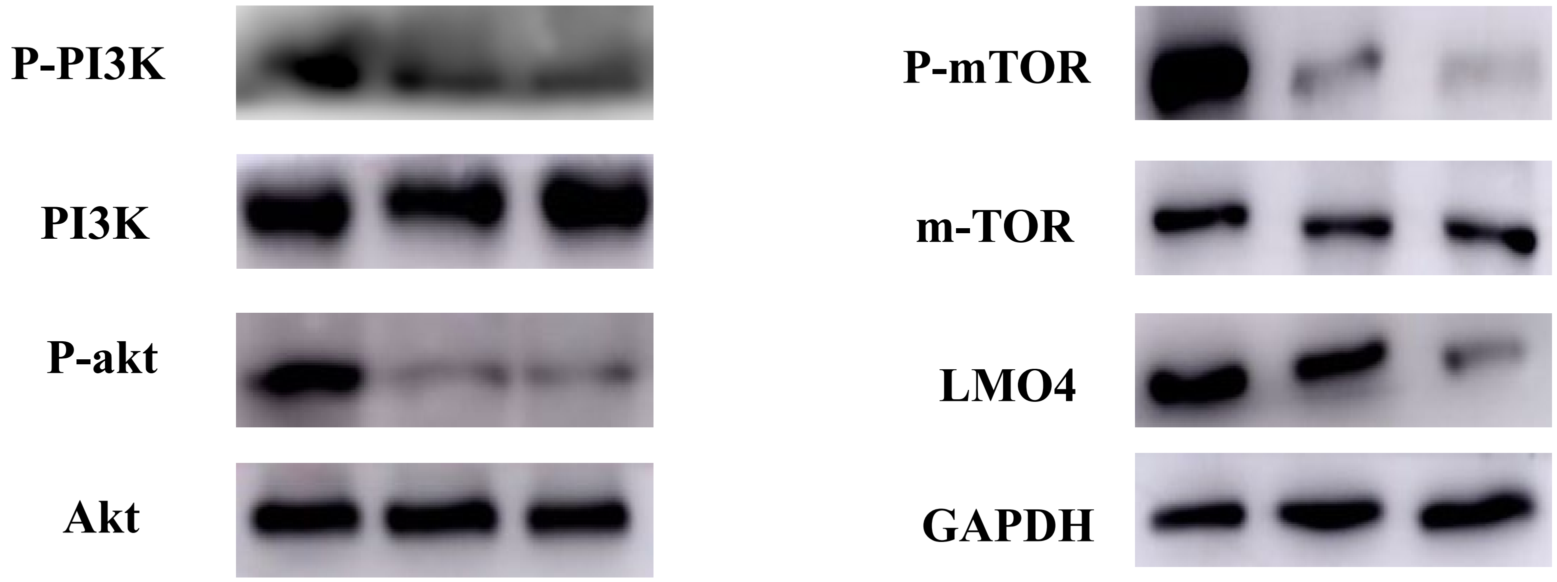

Full and uncropped western blot for Figure 6M

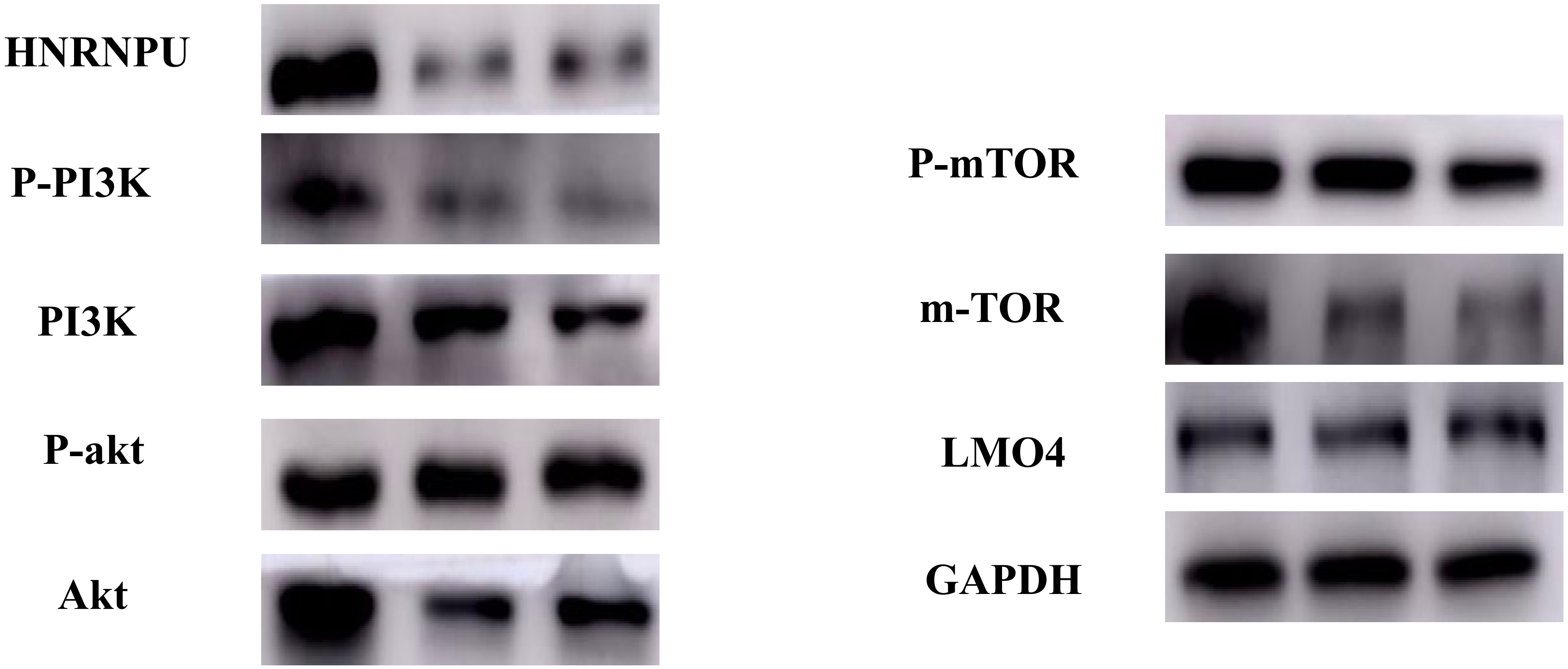

Full and uncropped western blot for Figure S1B

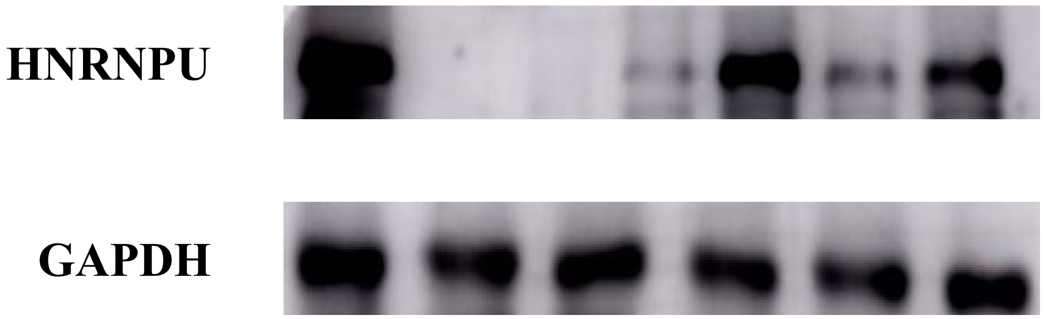

Full and uncropped western blot for Figure S5A

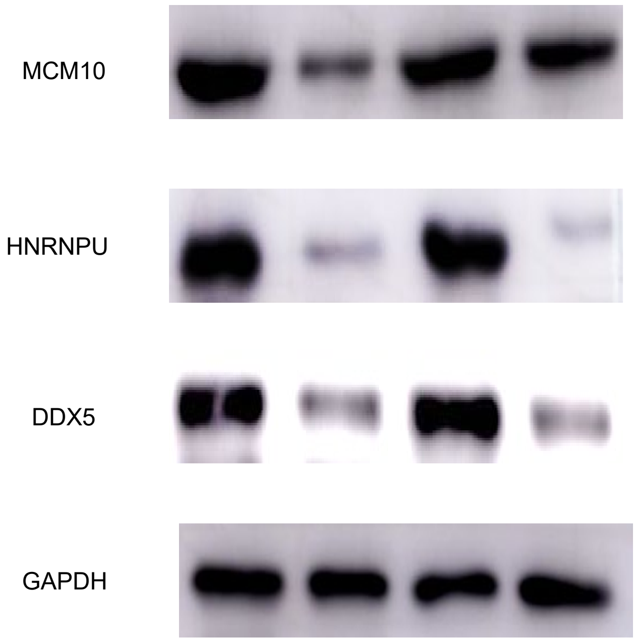

Full and uncropped western blot for Figure S5F

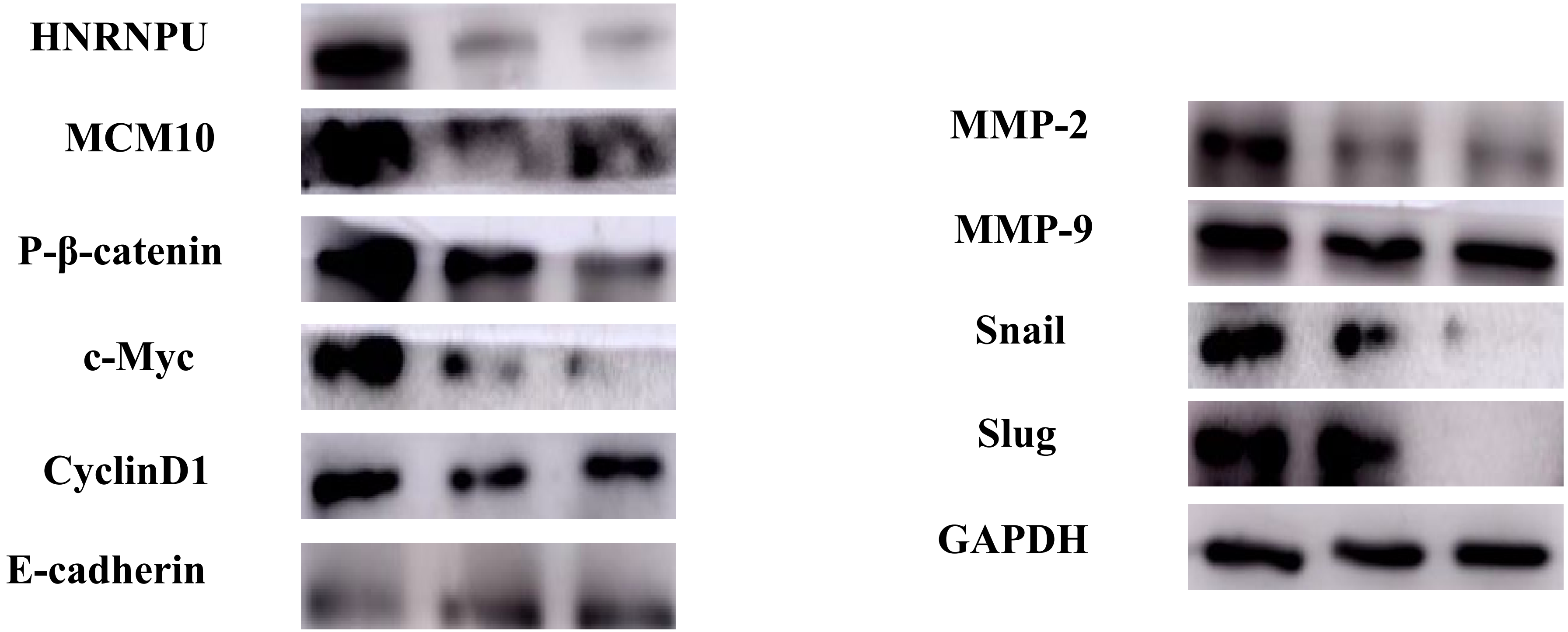

Full and uncropped western blot for Figure S5G

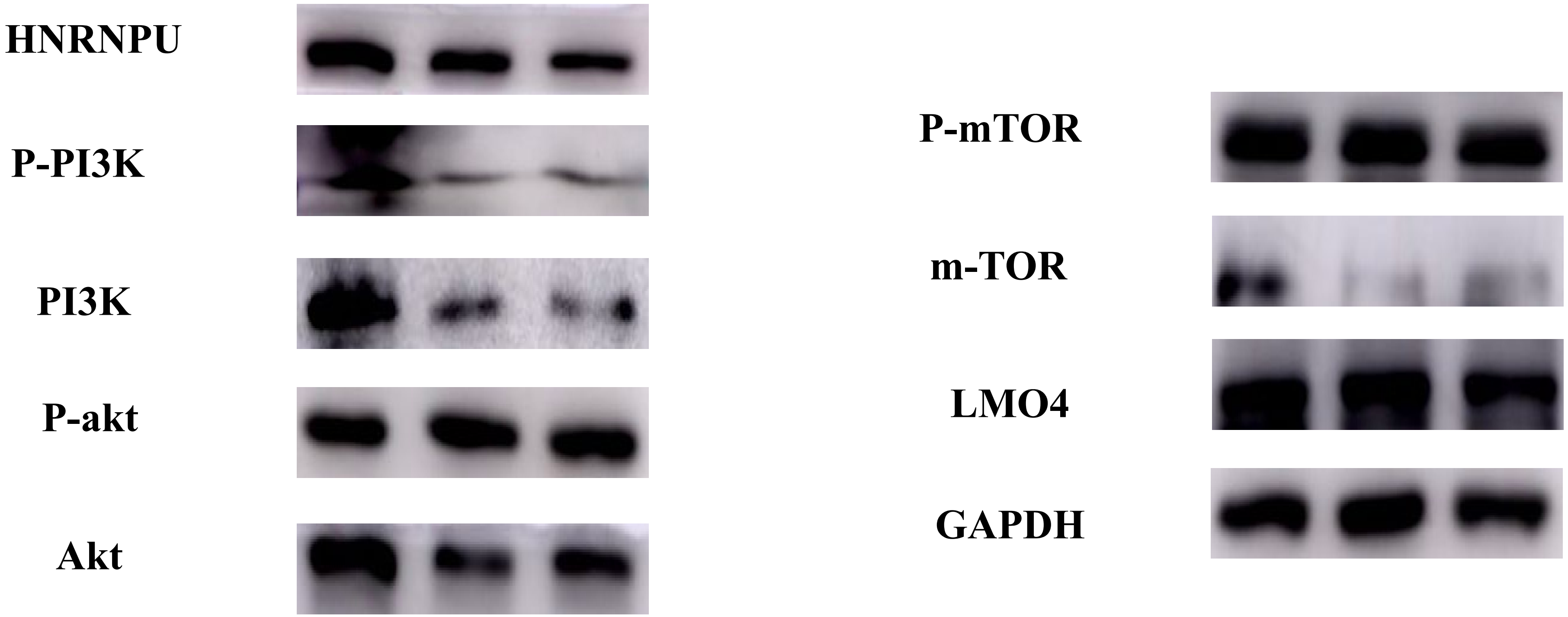

Supplement: Supplementary file 6 — Original Data File [file 41419_2022_5376_MOESM6_ESM.pdf]
